# Transmembrane and coiled-coil domain family 3 (TMCC3) regulates breast cancer stem cell and AKT activation

**DOI:** 10.1038/s41388-021-01729-1

**Published:** 2021-03-19

**Authors:** Ya-Hui Wang, Yu-Tzu Chan, Tsai-Hsien Hung, Jung-Tung Hung, Ming-Wei Kuo, Sheng-Hung Wang, Yenlin Huang, Yu-Ju Lin, Shin-Cheh Chen, Jyh-Cherng Yu, Jen-Chine Wu, John Yu, Alice L. Yu

**Affiliations:** 1 Institute of Stem Cell and Translational Cancer Research, Chang Gung Memorial Hospital at Linkou, Taoyuan, Taiwan; 2 Department of Anatomic Pathology, Chang Gung Memorial Hospital at Linkou, Taoyuan, Taiwan; 3 Chang Gung University, Taoyuan, Taiwan; 4 General Surgery Department, Chang Gung Memorial Hospital at Linkou, Taoyuan, Taiwan; 5 Division of General Surgery, Department of Surgery, Tri-Service General Hospital, National Defense Medical Center, Taipei, Taiwan; 6 Department of Pediatrics, University of California in San Diego, San Diego, CA USA

**Keywords:** Breast cancer, Oncogenes, Cancer stem cells

## Abstract

Cancer stem cells (CSC) play a pivotal role in cancer metastasis and resistance to therapy. Previously, we compared the phosphoproteomes of breast cancer stem cells (BCSCs) enriched subpopulation and non-BCSCs sorted from breast cancer patient-derived xenograft (PDX), and identified a function unknown protein, transmembrane and coiled-coil domain family 3 (TMCC3) to be a potential enrichment marker for BCSCs. We demonstrated greater expression of TMCC3 in BCSCs than non-BCSCs and higher expression of TMCC3 in metastatic lymph nodes and lungs than in primary tumor of breast cancer PDXs. TMCC3 silencing suppressed mammosphere formation, ALDH activity and cell migration in vitro, along with reduced tumorigenicity and metastasis in vivo. Mechanistically, we found that AKT activation was reduced by TMCC3 silencing, but enhanced by TMCC3 overexpression. We further demonstrated that TMCC3 interacted directly with AKT through its 1-153 a.a. domain by cell-free biochemical assay in vitro and co-immunoprecipitation and interaction domain mapping assays in vivo. Based on domain truncation studies, we showed that the AKT-interacting domain of TMCC3 was essential for TMCC3-induced AKT activation, self-renewal, and metastasis. Clinically, *TMCC3* mRNA expression in 202 breast cancer specimens as determined by qRT-PCR assay showed that higher *TMCC3* expression correlated with poorer clinical outcome of breast cancer, including early-stage breast cancer. Multivariable analysis identified *TMCC3* expression as an independent risk factor for survival. These findings suggest that TMCC3 is crucial for maintenance of BCSCs features through AKT regulation, and TMCC3 expression has independent prognostic significance in breast cancer. Thus, TMCC3 may serve as a new target for therapy directed against CSCs.

## Introduction

Breast cancer is the most common cancer among women worldwide. The majority of breast cancer deaths occur as a result of recurrent or metastatic disease rather than from the effects of the primary tumor [1]. The existences of cancer stem cells (CSCs) have been demonstrated in a variety of human cancers, including breast cancer [2, 3]. In 2003, Al-Hajj et al. reported that breast cancer stem cells (BCSCs) were enriched in the CD24^−^CD44^+^ subpopulation of breast cancer [2]. Subsequently, Ginestier et al. demonstrated that tumor cells with high aldehyde dehydrogenase activity (ALDH) which mediated the conversion of retinaldehyde to retinoic acid, harbored stem/progenitor properties [1, 4, 5]. These CSCs possessed the capacity for self-renewal, differentiation, and displayed resistance to chemotherapeutic agents and radiation, which might contribute to tumor relapse years after the clinical remission [6]. Thus, it will be important to understand the molecular characteristics of CSCs that may lead to the development of novel targets for CSC-directed therapy.

Mounting data have identified many dysregulated signaling pathways in BCSCs, including the JAK/STAT, *Hedgehog*, Wnt, Notch, PI3K/PTEN, and NF-κB [7–13]. In our previous study, we also demonstrated that IGF-1R/PI3K/AKT/mTOR pathway was crucial for BCSC maintenance [14]. In the pursuit of novel molecular signature of BCSCs, we performed comparative phosphoproteomic analysis of BCSCs and non-BCSCs [15]. Our interest in the novel protein, transmembrane and coiled-coil domain family 3 (TMCC3) was piqued by phosphoproteomic data showing greater phosphorylation of TMCC3 in BCSCs than in non-BCSCs.

Genes encoding putative proteins of the transmembrane and coiled-coil domain (TMCC) family have been found in many organisms. The TMCC family consists of three putative proteins (TMCC1–3) that are conserved from nematode to human [16]. However, the properties and functions of TMCC proteins are little known. It was reported that TMCC1 localizes to the rough ER and is crucial for ER-associated bud fission [16, 17]. TMCC2 is a neuronal, ER-located protein, and the interaction between TMCC2 and apoE contributes to amyloid-β protein precursor metabolism in Alzheimer’s disease [18]. Impaired function of dementin (*Drosophila* orthologue of TMCC2) causes neurodegeneration and early death in *Drosophila* [19]. TMCC3 is first cloned from human normal brain tissue. It is also predicted as an integral membrane protein and localized in ER [17, 20]. However, the function of TMCC3 remains elusive. In this study, we provide first evidence for the important roles of TMCC3 in self-renewal, metastasis, and tumorigenicity of BCSCs and AKT activation.

## Results

### BCSCs express higher levels of TMCC3 protein than non-BCSCs

**Fig. 1:**
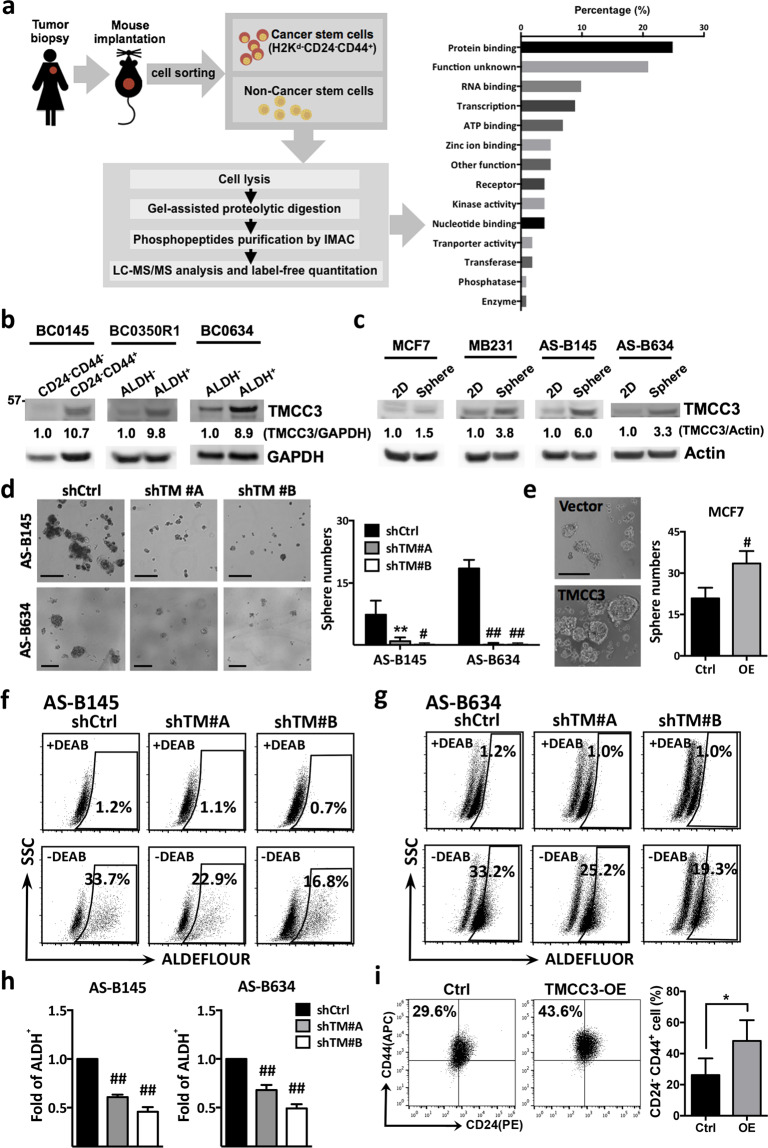
TMCC3 is highly expressed in BCSCs and crucial for BCSC maintenance. **a** Workflow illustration of phosphoproteomic analysis using BCSCs and non-BCSCs of BC0145 PDX tumor to discover function unknown phosphoproteins in BCSCs. **b** The protein levels of TMCC3 in BCSCs and non-BCSCs FACS-sorted from breast cancer PDX tumors, BC0145, BC0350R1, and BC0634 using H2k^d-^CD24^−^CD44^+^ (BC0145) and H2k^d−^ALDH^+^ (BC0350R1 and BC0634), respectively. **c** The protein levels of TMCC3 in MCF7, MDA-MB231 (MB231), AS-B145, and AS-B634 cultured as monolayer (2D) and mammosphere culture (Sphere) condition, as determined by western blotting. **d** The representative images of mammosphere in TMCC3 silenced AS-B145 and AS-B634 (left panels). Sphere forming capacities of AS-B145 and AS-B634 transduced with shRNA-TMCC3 (shTM #A and shTM #B) vs shRNA-control (shCtrl) after 7 days culture (right panels). Data represent mean ± SD. **p < 0.01, ^#^*p* < 0.001 and ^##^*p* < 0.0001 (*n* = 6, *t*-test). Scale bar = 200 μm. **e** The representative images of mammospheres and sphere forming capacities of MCF7 overexpressing TMCC3 vs vector control after 7 days culture. Data represent mean ± SD. ^#^*p* < 0.001 (*n* = 6, *t*-test). Scale bar = 200 μm. **f**, **g** ALDH activity of TMCC3 silenced AS-B145 and AS-B634 (shTM #A and shTM #B), as compared with their respective controls. ALDH^+^ subpopulation was determined by flow cytometry and the relative folds of ALDH^+^ cells were calculated from three independent experiments (**h**). Data represent mean ± SEM. ^##^*p* < 0.0001 (*n* = 3, *t*-test). **i** CD24^−^CD44^+^ cell population of TMCC3-overexpressed MCF7 was determined by flow cytometry, as compared with vector control transduced MCF7. Data represent mean ± SEM. *p = 0.01 (*n* = 6, *t*-test).

Previously, we established several patient-derived xenograft tumors (PDXs) of breast cancer and identified their enrichment markers for BCSCs by determining the frequency of tumor-initiating cells, after sorting by fluorescence-activated cell sorter. Mice were injected with serial dilution of sorted cells to identify the markers for enrichment of BCSC. The supporting evidence for CD24^−^CD44^+^ as BCSC enrichment marker for BC0145 was published previously [14]. In our previous phosphoproteomic analysis of BCSCs and non-BCSCs sorted from BC0145 PDX, the functions of ~21% of 455 phosphoproteins upregulated in BCSCs, including TMCC3, were unknown (Fig. 1a) [15]. In replicate phosphoproteomic studies, greater phosphorylated TMCC3 at Serine 216 was noted in BCSCs than in non-BCSCs by 1.5 and 3.8 folds (Supplementary Fig. S1a). TMCC3 belongs to the TMCC family that includes TMCC1–3 and contains two coiled-coil domains of the N-terminal region and two transmembrane domains of the C-terminal region (Supplementary Fig. S1b). Sequence alignment of TMCC3 from various vertebrate species shows several highly conserved regions (Supplementary Fig. S1c), suggesting the important roles of this protein in the vertebrates.

To confirm the differential expression levels of TMCC3 protein in BCSCs enriched populations and non-BCSCs, we determined BCSC enrichment markers in three breast cancer PDXs. Previously, H2k^d−^CD24^−^CD44^+^ cell population was shown to be enriched in BCSC of BC0145 PDX [14]. For BC0350R1 and BC0634 PDXs, aldehyde dehydrogenase (H2k^d−^ALDH^h^) activity was characterized as the BCSC enrichment marker base on tumorigenicity assay in vivo (Supplementary Table S1). Using the above-identified BCSC enrichment markers, BCSCs and non-BCSCs were sorted from these three PDXs for western blotting. As shown in Fig. 1b, the protein levels of TMCC3 were higher in BCSCs than non-BCSCs by 10.7, 9.8, and 8.9 folds, for BC0145, BC0350R1, and BC0634 tumors, respectively.

To facilitate in vitro investigation of the involvement of TMCC3 in BCSCs, AS-B145 and AS-B634 cells were cultured from sorted H2K^d−^CD24^−^CD44^+^ cells of BC0145 and H2K^d−^ALDH^h^ cells of BC0634 PDXs, respectively, for limited passages (≤10 passages) [14, 15]. Using these two short-term cultured cells and established breast cancer cell lines, we compared the expression levels of TMCC3 in mammosphere-cultured (Sphere) and monolayer-cultured (2D) cells. As shown in Fig. 1c, TMCC3 protein expression was greater in mammosphere-cultured cells by 1.5, 3.8, 6.0, and 3.3 folds in MCF7, MDA-MB231 (MB231), AS-B145, and AS-B634, respectively. These findings suggest that TMCC3 may play a role in the maintenance of BCSCs.

### TMCC3 contributes to mammosphere formation and ALDH activity in BCSCs

To delineate the roles of TMCC3 in BCSCs, we examined the effects of changing TMCC3 expression on the mammosphere formation and ALDH activity which are important features of BCSCs [1, 2, 21–23] by lentivirus-mediated silencing or overexpression. Upon silencing of TMCC3 by shRNA #A, #B, or #C clones, *TMCC3* mRNAs were reduced to 0.24 ± 0.02, 0.09 ± 0.02, and 0.3 ± 0.01 folds, respectively, of control shRNA transfected AS-B145 cells; and to 0.42 ± 0.02, 0.23 ± 0.06, and 0.82 ± 0.12 folds, respectively, in AS-B634 (Supplementary Fig. S2a). Western blotting confirmed the reduction of TMCC3 protein to negligible levels (Supplementary Fig. S2b). shRNA-TMCC3 #A and #B clones, which provided better knockdown efficiency were chosen for further studies. Stable clone of TMCC3 overexpression was established using MCF7, which expressed low level of TMCC3 (Supplementary Fig. S2c, d). As shown in Fig. 1d, mammosphere numbers were significantly lower in shRNA-TMCC3 #A and #B transduced AS-B145 (1.0 ± 0.4 and 0.2 ± 0.2) and AS-B634 (0.2 ± 0.2 and 0.2 ± 0.2) than in shRNA-control (7.4 ± 1.5 and 18.5 ± 0.8, *n* = 6, *p* < 0.0001). To confirm the participation of TMCC3 in mammosphere formation, we also determined the mammosphere forming capacity of TMCC3 overexpressing MCF7. As shown in Fig. 1e, greater mammosphere number was observed in TMCC3 overexpressing cells (33.5 ± 4.5), as compared with the vector control cells (22.5 ± 2.9, *n* = 6, *p* < 0.001). In addition, ALDH^+^ subpopulation of AS-B145 was reduced from 33.7% (shControl) to 22.9% (shTM #A) and 16.8% (shTM #B) upon TMCC3 silencing (Fig. 1f, h), and the relative folds of ALDH^+^ subpopulation of AS-B145 was significantly reduced by shTM #A and #B to 0.61 ± 0.01 (*n* = 3, *p* < 0.0001) and 0.46 ± 0.03 (*n* = 3, *p* < 0.0001) folds, respectively, of shControl. Similarly, ALDH^+^ subpopulation of AS-B634 was significantly reduced by shTM #A and shTM #B to 0.68 ± 0.03 (*n* = 3, *p* < 0.0001) and 0.49 ± 0.02 (*n* = 3, *p* < 0.0001) folds, respectively, of shControl (Fig. 1g, h). On the other hand, upon TMCC3 overexpression in MCF7, the CD24^−^CD44^+^ subset which has been reported to enrich BCSCs increased from 26.15 ± 4.38% to 48.17 ± 5.46% (*n* = 6, *p* = 0.01) (Fig. 1i) [24]. These findings indicate that TMCC3 is crucial for sphere formation, ALDH activity, and CSC enrichment, which are important features of BCSCs.

### TMCC3 silencing suppresses tumor growth and tumorigenesis in vivo

**Fig. 2:**
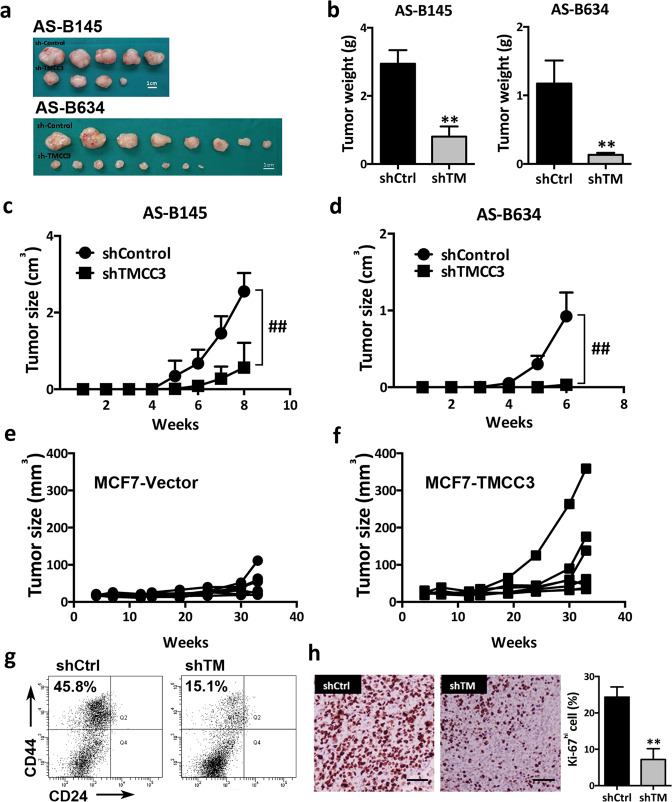
TMCC3 is essential for tumorigenicity in vivo. **a**, **b** TMCC3 silenced AS-B145, AS-B634, and their control cells were implanted into mammary fat pad of NSG female mice and tumor sizes were monitored weekly. The photographs (**a**) and weights (**b**) of tumors from control (shControl) and TMCC3 silenced (shTMCC3) AS-B145 and AS-B634. **c**, **d** Tumor growth curves of control and TMCC3 silenced AS-B145 and AS-B634. Data represent mean ± SD (*n* = 5, AS-B145 and *n* = 8, AS-B634). **e**, **f** Tumor growth curve of TMCC3-overexpressed and vector transduced MCF7. **g** Effects of TMCC3 silencing on breast cancer stem cell population (H2k^d−^CD24^−^CD44^+^) of AS-B145 PDX tumor as determined by flow cytometry. **h** The expression of Ki67 in shControl and shTMCC3 transduced AS-B634 PDX tumors (*n* = 3). Scale bar = 100 μm. **p < 0.01, ^##^*p* < 0.0001.

We next evaluated the in vivo effects of TMCC3 silencing on the tumorigenicity of AS-B145 and AS-B634. Control shRNA (shControl) and shRNA-TMCC3 (shTMCC3 #B) transduced AS-B145 and AS-B634 were injected into mammary fat pads of NSG female mice. Tumor size was monitored weekly for 6–8 weeks, and tumors were harvested for BCSC population examination and Ki67 staining. As shown in Fig. 2, the photographs (a) and weights (b) of tumors from TMCC3 silenced (shTMCC3) AS-B145 and AS-B634 were obviously smaller than control tumors (shControl) (*p* < 0.01). The growth rates of TMCC3 silenced tumors were also significantly slower than control tumors (*p* < 0.0001 for both AS-B145 and AS-B634) (Fig. 2c, d). We also examined the tumor growth of TMCC3 overexpressing MCF7 in vivo. As shown in Fig. 2e, f, TMCC3 overexpressing tumors (Fig. 2f) grew faster than the vector control group (Fig. 2e). Analysis of BCSC cell population (H2k^d−^CD24^−^CD44^+^) in the TMCC3 silenced AS-B145 tumors showed reduction of BCSCs from 45.8% in shControl tumors to 15.1% in shTMCC3 tumors (Fig. 2g). Furthermore, Ki67 expression was significantly reduced from 24.3 ± 1.6% in shControl AS-B634 tumors to 7.2 ± 1.7% in TMCC3 silenced tumors (*n* = 3, *p* < 0.01) (Fig. 2h). These findings indicate that TMCC3 is not only important for tumor growth, but also crucial for the maintenance of BCSC population in vivo.

To determine the tumor-initiating capacity (TIC) of TMCC3 silenced AS-B145 and AS-B634, serial dilutions of cells were injected into NSG mice. As shown in Table 1, TIC of AS-B145 and AS-B634 decreased from 1:19,707 to 1:70,682 (*p* = 0.014) and 1:59 to 1:987 (*p* < 0.001), respectively, after TMCC3 knockdown. This provides further evidence for the crucial role of TMCC3 in CSC properties in vivo.

**Table 1:** TMCC3 silencing of AS-B145 and AS-B634 reduces their tumor-initiating capacities^a^.

		(Cell number of injection)						Stem cell frequency	*p* value
		10^5^	5 × 10^4^	10^4^	10^3^	10^2^	50		
AS-B145	shControl	5/5	5/6	3/5				1:19,707	0.014
	shTMCC3	4/5	3/7	1/5				1:70,682	
AS-B634	shControl			8/8	5/5	4/5	3/5	1:59	<0.001
	shTMCC3			8/8	3/5	1/5	0/5	1:987	

^a^BCSC-enriched population of AS-B145 and AS-B634 were infected with shTMCC3 #B or shControl and injected into mammary fat pads of NSG mice.

### TMCC3 is crucial for tumor metastasis in vitro and in vivo

**Fig. 3:**
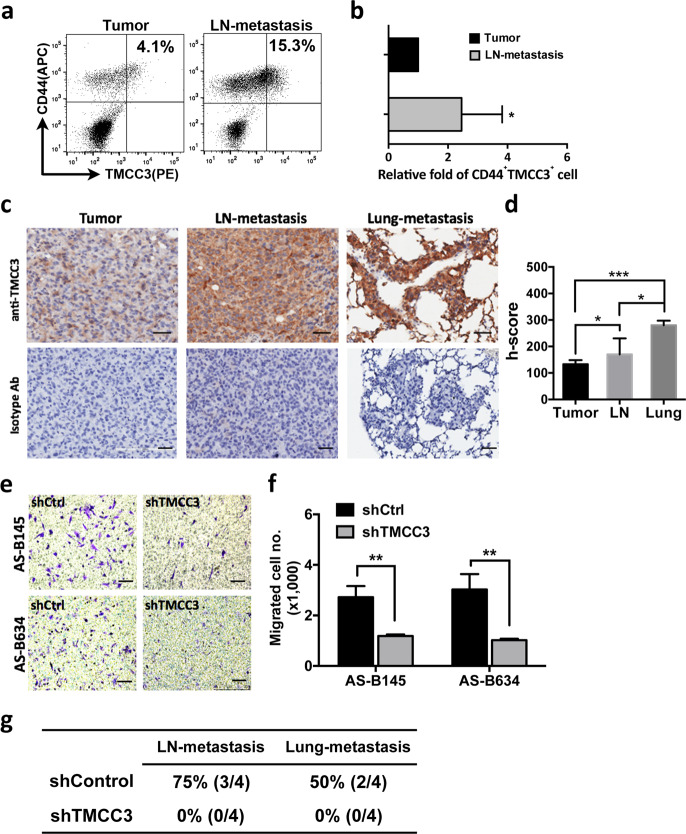
TMCC3 contributes to tumor metastasis in vitro and in vivo. **a** FACS analysis of TMCC3 expression in permeabilized BCSC (CD44^+^) harvested from primary tumor (Tumor) and metastatic lymph node (LN-metastasis) of BC0145 PDX tumor. **b** Percent of CD44^+^TMCC3^+^ cell population in metastatic lymph node was normalized to that in primary tumor to show the relative folds. *p < 0.05 (*n* = 5, *t*-test). **c** The representative IHC stainings of primary tumor, lymph node, and lung-metastatic tissues from one of three BC0634 bearing mice. Scale bar = 100 μm. **d** The histoscores (h-score) of TMCC3 expression in tumors of three mice were calculated. *p < 0.05, ***p < 0.001 (*n* = 3, *t*-test). **e**, **f** The numbers of migrated cells in each group were determined in trans-well migration assay. **p < 0.01 (*n* = 3, *t*-test). Scale bar = 100 μm. **g** Metastatic frequency of TMCC3 silenced AS-B634. 2 × 10^4^ shTMCC3 or shControl transduced AS-B634 were implanted into mammary fat pad of NSG mice (*n* = 4). Tumor, lymph node, and lung of tumor bearing mice were harvested at 3 months after implantation.

The propensity of CSCs for tumor metastasis is well documented [25, 26]. To delineate the involvement of TMCC3 in metastasis of breast cancer, we examined the expression of TMCC3 in metastatic lesions of breast cancer PDXs. Two to three months after injection of BC0145 or BC0634 tumor cells in mammary fat pads of NSG mice, metastatic lesions were observed in ipsilateral lymph nodes and lungs. The lymph nodes and primary tumor of BC0145 PDX were harvested for staining with anti-CD44 and anti-TMCC3 antibodies. As shown in Fig. 3a, 4.1% and 15.3% of cells in the BC0145 primary tumor and metastatic lymph node, respectively, were CD44^+^TMCC3^+^. The relative fold of CD44^+^TMCC3^+^ subsets in metastatic lymph node was 2.72 ± 0.7, as compared with primary tumor cells (Fig. 3b, *n* = 5, *p* = 0.04). In addition, the representative IHC staining for TMCC3 expression of primary tumor, lymph node, and lung-metastatic tissues from one of three BC0634 bearing mice were shown in Fig. 3c. The histoscores (h-score) of TMCC3 expression in tumors as shown in Fig. 3d demonstrated highest TMCC3 expression in lung lesions followed by lymph node and then primary tumors (*n* = 3, *p* < 0.05). These findings suggest that TMCC3 may contribute to tumor metastasis.

To examine the role of TMCC3 in tumor metastasis, we next determined whether TMCC3 silencing suppresses the migration of TMCC3 expressing BCSCs in migration assay. TMCC3 silencing of AS-B145 and AS-B634 significantly reduced the migrated cell numbers to 1.19 ± 0.03 and 1.02 ± 0.03 (×1000) cells/well, respectively, as compared to 2.72 ± 0.26 and 3.03 ± 0.35 (×1000) cells/well, respectively, in control cells (Fig. 3e, f, *n* = 3, *p* < 0.01).

To provide further evidence supporting the notion that TMCC3 is important for metastasis, we examined the impact of TMCC3 silencing on the metastatic frequency of AS-B634. After injection of AS-B634, primary tumor, lymph node, and lung of tumor bearing mice were harvested at 3 months. As shown in Fig. 3g, we found TMCC3 silencing abolished metastasis in vivo. The lymph node and lung-metastatic frequency were 75% (3/4) and 50% (2/4), respectively, in control group, but none in TMCC3 knockdown. These findings support the participation of TMCC3 in tumor metastasis in vitro and in vivo.

### TMCC3 is involved in AKT activation in BCSCs

**Fig. 4:**
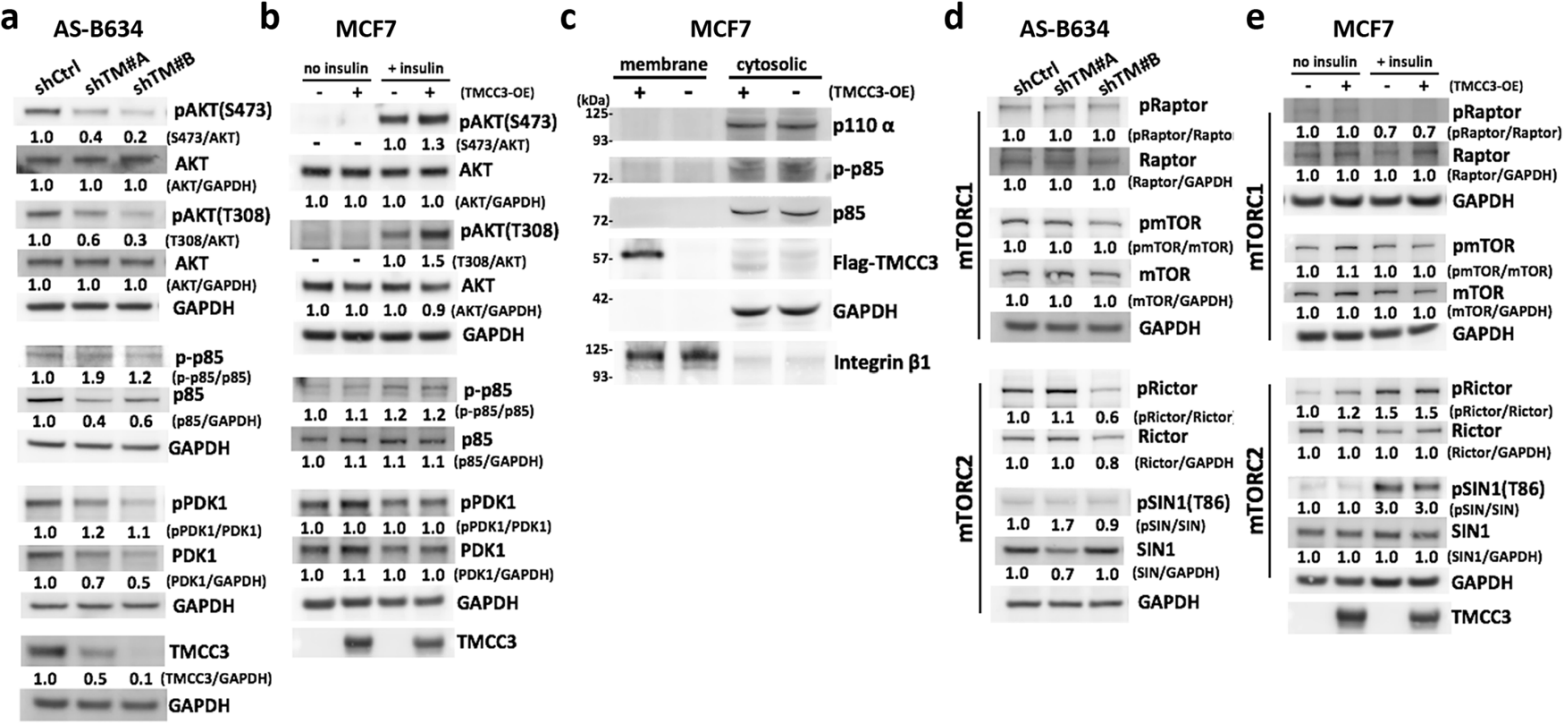
TMCC3-induced AKT activation likely does not involve PI3K/PDK1/mTORC1 or mTORC2 pathway. AS-B634 cells were transduced with shRNA-TMCC3 #A, #B, or shRNA-control for 3 days. MCF7 cells were transiently transfected with TMCC3 in the presence (+) or absence (−) of 10 μg/ml human insulin for 5 min after 12 h serum starvation. Levels of phosphorylated AKT (S473 and T308), p85 of PI3K and PDK1, and total protein in TMCC3 silenced AS-B634 (**a**) and TMCC3 overexpressing MCF7 (**b**), were determined by western blotting. Relative intensity was quantified and normalized to non-phospho protein or GAPDH. **c** Subunit p85 phosphorylation and p110-α membrane translocation were determined in TMCC3 overexpressing MCF7 cells. Integrin β1 and GAPDH served as membrane and cytoplasmic markers, respectively. **d**, **e** mTORC1 (Raptor and mTOR) and mTORC2 (Rictor and SIN1) activation were assessed in TMCC3 silenced AS-B634 and TMCC3 overexpressing MCF7 cells.

It is well known that AKT plays important roles in the center of diverse signaling cascades and essential to stem cell activation, CSC survival, and self-renewal [27–32]. Previously, we demonstrated that IGF-1R/PI3K/AKT/mTOR pathway was important for BCSC maintenance [14]. To examine a possible linkage of TMCC3 to AKT signaling, we determined AKT phosphorylation in TMCC3 silenced AS-B634. As shown in Fig. 4a, TMCC3 knockdown by shTM #A and #B decreased the relative folds of pAKT^S473^/AKT to 0.4 and 0.2, and pAKT^T308^/AKT to 0.6 and 0.3 in AS-B634, respectively. On the other hand, relative folds of pAKT^S473^ and pAKT^T308^ increased to 1.3 and 1.5 in TMCC3 overexpressing MCF7 upon insulin stimulation, respectively (Fig. 4b). These findings suggest that AKT activation may contribute to the critical role of TMCC3 in maintenance of BCSC properties. We further examined the possibility that TMCC3 may enhance AKT phosphorylation via activation of PI3K/PDK1, the up-stream regulators of AKT. As shown in Fig. 4a, b and Supplementary Fig. S3, the phosphorylation of regulatory subunit p85 (p-p85/p85) of PI3K and PDK1 (pPDK/PDK) did not decrease upon TMCC3 silencing in AS-B634 or increase in TMCC3 overexpressing MCF7. In addition, TMCC3 overexpression in MCF7 did not enhance membrane translocation of catalytic subunit p110-α of PI3K (Fig. 4c). These findings suggested that TMCC3-induced AKT activation likely does not involve PI3K or PDK1 phosphorylation.

Recent studies have identified that mTOR complex 1 (mTORC1) and mTORC2 activation correlate to AKT phosphorylation [33–35]. To explore the consequence of TMCC3-induced AKT activation, we examined mTORC1 and mTORC2 phosphorylation upon TMCC3 knockdown or overexpression. As shown in Fig. 4d, e, there were no alterations in the activation of mTORC1 (pRaptor/Raptor and p-mTOR/mTOR) and mTORC2 (pRictor/Rictor and p-SIN1/SIN1) in TMCC3 silenced or overexpressing cells. However, the use of phosphoproteins/total proteins ratios to reflect activation status were affected by reduction of total p85 and PDK1 proteins in shTM#A and shTM#B transduced AS-B634 (Fig. 4a), and total Rictor and SIN1 proteins in one of shRNA-TMCC3 transduced cells (Fig. 4d). It should be noted that TMCC3 silencing reduced cell growth (Supplementary Fig. S4), which may explain the decrease in these signaling proteins. Taken together, these findings suggested that PI3K, PDK1, mTORC1, or mTORC2 signalings are most likely not involved in TMCC3-induced AKT activation.

### 1-153 a.a. domain of TMCC3 contributes to interaction and activation of AKT

**Fig. 5:**
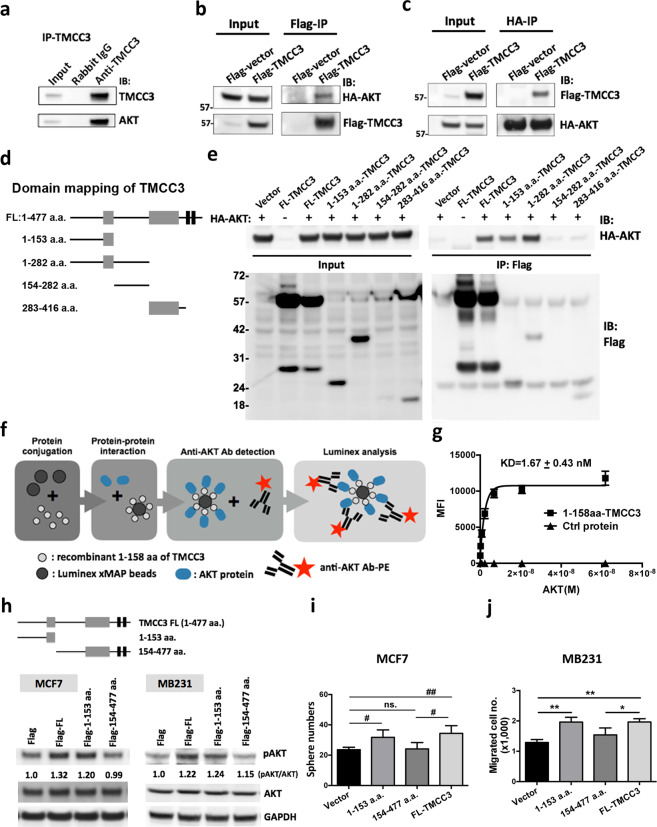
1-153 a.a. domain of TMCC3 directly interacts with AKT and enhances AKT activation. **a** Presence of AKT in immunoprecipitate of TMCC3 from AS-B634 using anti-TMCC3 antibody. **b**, **c** MDA-MB231 cells transfected with flag-tagged full-length (FL)-TMCC3 and HA-tagged AKT were subject to co-immunoprecipitation assay using anti-flag antibody (**b**) or anti-HA antibody (**c**), followed by immunoblotting. **d** Diagram of full-length TMCC3 and truncated forms of TMCC3 containing 1-153 a.a., 1-282 a.a., 154-282 a.a., 283-416 a.a. **e** Co-immunoprecipitation analysis of flag-tagged TMCC3 with HA-tagged AKT proteins. 293T cells were transfected with a plasmid encoding HA-tagged AKT and another plasmid encoding flag tag alone or various flag-tagged TMCC3 forms: full-length (FL), 1-153 a.a., 1-282 a.a., 154-282 a.a., or 283-416 a.a. **f** Flowchart of luminex immunosandwich assay. Detailed procedures were described in “Materials and methods”. **g** Mean fluorescence intensities (MFI) showed AKT bound to beads coupled with 1-158 a.a. domain of TMCC3 proteins (*n* = 4). **h** Levels of pAKT^S473^ and total AKT were examined in flag-tagged full-length (Flag-FL), 1-153 a.a.- or 154-477 a.a.-truncated TMCC3 transfected MCF7 and MDA-MB231 (MB231) by western blot analysis. Mammosphere forming capacities (**i**) and migration abilities (**j**) were assessed in cells transfected with flag-tagged full-length (Flag-FL), 1-153 a.a.- or 154-477 a.a.-truncated TMCC3. **i** Sphere numbers were calculated after culture for 7 days. ^#^*p* < 0.001, ^##^*p* < 0.0001 (*n* = 8, *t*-test). **j** The numbers of migrated cells in each group were determined in trans-well migration assay. *p < 0.05, **p < 0.01 (*n* = 3, *t*-test).

In order to ascertain the regulation of TMCC3 on AKT activation, we pulled down the endogenous TMCC3 in AS-B634 with anti-TMCC3 antibody. As shown in Fig. 5a, AKT was detected in the immunoprecipitation. To confirm the interaction of TMCC3 and AKT, we constructed flag-tagged TMCC3 and HA-tagged AKT for co-immunoprecipitation assay. As shown in Fig. 5b, c, HA-tagged AKT was pulled down with flag-tagged TMCC3 using anti-flag antibody, and vice versa.

To decipher the AKT-interacting domain of TMCC3, we constructed flag-tagged full-length (1-477), 1-153, 1-282, 154-282, and 283-416 amino acid (a.a.) domains of TMCC3 for immunoprecipitation assay (Fig. 5d). 293T cells were co-transfected with flag-tagged TMCC3 (full-length or truncated protein) and HA-tagged AKT. After flag protein pull-down using anti-flag antibody, the immunoprecipitates were examined for HA-AKT proteins with anti-HA antibody. As shown in Fig. 5e, AKT was pulled down together with 1-153, 1-282 a.a. domains and full-length TMCC3, but not with 283-416 a.a. domain. However, the expression of 154-282 a.a. domain of TMCC3 was too low to delineate whether it interacted with AKT or not. To provide evidence for direct interaction between TMCC3 and AKT in a cell-free system, luminex immunosandwich assay was performed as illustrated in Fig. 5f [36]. Recombinant 1-158 a.a. domain of TMCC3 protein was generated and coupled onto Luminex beads, which were then incubated with various concentrations of recombinant AKT. After washing, AKT interacting with bead-bound 1-158 a.a. domain of TMCC3 were recognized by the fluorescence labeled anti-AKT antibody. As shown in Fig. 5g, AKT can bind directly with 1-158 a.a. domain of TMCC3 protein in vitro in a concentration dependent manner, with KD of 1.67 ± 0.43 nM. On the other hand, AKT did not bind to bead-bound control protein (Puf-A) [37]. These findings support the notion that 1-158 a.a. domain of TMCC3 directly interacted with AKT without other accessory proteins.

To delineate whether interaction of TMCC3 with AKT contributes to TMCC3-induced AKT activation, we generated TMCC3 constructs containing truncation of 1-153 a.a. or 154-477 a.a.. MCF7 and MDA-MB231 (MB231) cells were transfected with flag-tagged full-length, 1-153 a.a.-, 154-477 a.a.-truncated TMCC3 or vector control (Supplementary Fig. S5), and pAKT^S473^ and total AKT were determined. As shown in Fig. 5h, pAKT^S473^ was upregulated to 1.32 and 1.20 folds of vector control in MCF7 transfected with full-length or 1-153 a.a.-truncated TMCC3, respectively. But no change in cell transfected with 154-477 a.a.-truncated TMCC3. Similar results were also observed in MB231 cells. pAKT^S473^ was increased to 1.22 and 1.24 folds of vector control in cells transfected with full-length or 1-153 a.a.-truncated TMCC3, respectively. These findings suggest that 1-153 a.a. domain of TMCC3 which interacted directly with AKT is crucial for AKT activation. In addition, we further examined the contribution of AKT-interacting region of TMCC3 to mammosphere formation and migration ability. MCF7 and MDA-MB231 were transfected with flag-tagged full-length, 1-153 a.a.-, 154-477 a.a.-truncated TMCC3 or vector control. As shown in Fig. 5i, mammosphere formation was significantly enhanced in cells transfected with 1-153 a.a. domain (31.75 ± 1.74, *n* = 8, *p* < 0.001) or FL-TMCC3 (34.38 ± 1.80, *p* < 0.0001), as compared with vector control cells (23.63 ± 0.57), but not in 154-477 a.a. domain of TMCC3 expressing cells (24.13 ± 1.48). Next, we examined the migration ability of truncated or full-length TMCC3 expressing cells. As shown in Fig. 5j, the migration capacity was significantly improved in cells expressing 1-153 a.a. domain or FL-TMCC3, as compared with control cells (*n* = 3, *p* < 0.01 and *p* < 0.01, respectively), but not in 154-477 a.a. domain of TMCC3 transfected cells. Our findings support that AKT-interacting domain (1-153 a.a.) of TMCC3 could enhance AKT activation and contribute to BCSC properties.

### Expression of *TMCC3* mRNA in breast cancer tissues

To evaluate the clinical relevance of *TMCC3* expression, we examined the mRNA levels of *TMCC3* in tumor specimens of 202 patients with breast cancer by qRT-PCR. Clinical characteristics and demographic information of these patients were summarized in Supplementary Table S2. The mean age was 54.4 ± 12.2 (range: 30–89). The median follow-up time was 9.55 years (range: 0.30–12.7 years). The relationship between *TMCC3* mRNA expression and clinical pathological variables in breast cancer was presented in Supplementary Table S3. Among 202 patients, 51 patients (25.7%) had disease recurrence. Using Youden index to determine the optimal cutoff values defining high and low expression groups, we found that patients with high expression level of tumor *TMCC3* mRNA were at greater risk than those with low expression level for lymph nodes involvement (*p* = 0.04, OR: 1.89, 95% CI: 1.06–3.42), relapse (*p* < 0.001, OR: 4.45, 95% CI: 2.27–8.69), and death (*p* < 0.001, OR: 3.77, 95% CI: 1.96–7.23) (Supplementary Table S3).

### Association of *TMCC3* expression levels with survival

**Fig. 6:**
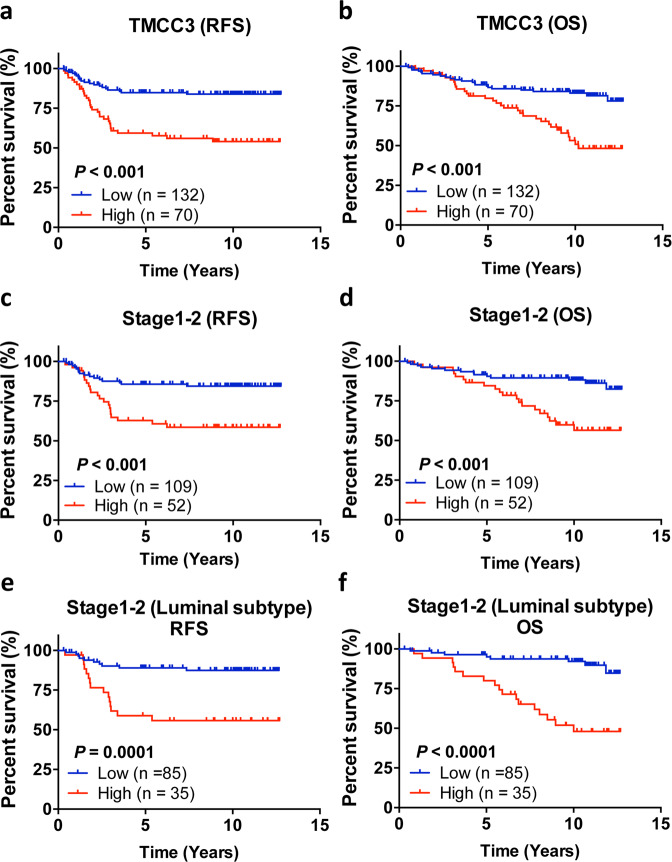
High expression of *TMCC3* correlates with poor clinical outcome of breast cancer patients. **a**, **b** The mRNA levels of *TMCC3* in tumor specimens of 202 patients with breast cancer were analyzed by qRT-PCR. Kaplan–Meier plots of relapse-free survival (RFS) and overall survival (OS) for patients in relation to *TMCC3* mRNA levels, and using Youden index to determine the optimal cutoff values defining high (red) and low (blue) expression groups. High and low expression of *TMCC3* in relation to RSF (**a**) and OS (**b**) were shown in tumor tissue (*n* = 202) with breast cancer. High and low expression of *TMCC3* in relation to RSF (**c**) and OS (**d**) were shown in stages 1–2 breast cancer tumor tissue (*n* = 161 for stages 1–2 breast cancers). In stages 1–2 luminal A + B breast cancers, high and low expression of *TMCC3* in relation to RSF (**e**) and OS (**f**) were shown in tumor tissue (*n* = 120 for stages 1–2 luminal A + B breast cancers). Survival curves were plotted with Kaplan–Meier method, by the log-rank test applied for comparison. The Cox proportional-hazards regression model was employed to evaluate the independent prognostic factors. The statistical analyses were performed with Prism 5.0 (GraphPad Software, La Jolla) and SPSS ver. 22.0 (IBM, Armonk) software.

The relapse-free survival (RFS) and overall survival (OS) of breast cancer patients as analyzed by the Kaplan–Meier method, and a log-rank test showed that patients with low expression level of *TMCC3* in tumor had significantly greater RFS (*p* < 0.001) and OS (*p* < 0.001) than patients with high expression levels (Fig. 6a, b). We then analyzed the potential prognostic value of *TMCC3* expression in 161 patients with early-stage disease (stages 1–2). As shown in Fig. 6c, d, early-stage patients with low expression of *TMCC3* in tumor part had significantly greater RFS (*p* < 0.001) and OS (*p* < 0.001) than patients with high expression levels. The survival benefit of patients with low expression of *TMCC3* was even more striking for early-stage luminal A and B patients (*p* = 0.0001 for RFS, *p* < 0.0001 for OS) (Fig. 6e, f). These results demonstrated the adverse impacts of high expression of *TMCC3* on clinical outcome of breast cancer, especially for those with early-stage luminal subtypes.

### Higher expression level of *TMCC3* is an independent prognostic factor for breast cancer

To evaluate the potential value of *TMCC3* expression levels for predicting RFS and OS of breast cancer, univariate Cox proportional hazard regression analyses were conducted. The results indicated that RFS correlated with patients aged ≥50 years (HR: 2.74, 95% CI: 1.43–5.24, *p* = 0.002) and Grade III (HR: 1.89, 95% CI: 1.04–3.46, *p* = 0.038), and higher expression of *TMCC3* in tumor (HR: 3.32, 95% CI: 1.89–5.83, *p* = 0.001) (Table 2). In addition, OS correlated with the patients aged ≥50 years (HR: 2.66, 95% CI: 1.42–4.96, *p* = 0.002), Grade III (HR: 1.89, 95% CI: 1.06–3.40, *p* = 0.032), stages III + IV (HR: 2.81, 95% CI: 1.62–4.89, *p* = 0.001) and higher expression of *TMCC3* in tumor (HR: 2.92, 95% CI: 1.69–5.02, *p* = 0.001) (Table 2). Next, to identify the independent variables associated with poor RFS or OS, we selected those important covariates that showed statistical significance in the univariate analysis for multivariable Cox regression analysis in a stepwise manner. As shown in Table 2, age was independent risk factors for RFS. And age and stage were independent risk factors for OS. Notably, the expression level of tumor *TMCC3* was an independent risk factor for RFS (HR: 2.81, 95% CI: 1.45–5.45, *p* = 0.002) and OS (HR: 2.28, 95% CI: 1.31–3.96, *p* = 0.004). These data indicate that *TMCC3* expression in tumor tissue is an important and independent predictor for RFS and OS in breast cancer patients.

**Table 2:** Association of risk factors with recurrence and survival of breast cancer in univariate and multivariable analysis.

Variables	RFS						OS					
	Univariate analysis			Multivariable analysis			Univariate analysis			Multivariable analysis		
	HR	95% CI	*p* value	HR	95% CI	*p* value	HR	95% CI	*p* value	HR	95% CI	*p* value
Age: ≥50 vs <50	2.74	1.43–5.24	**0.002**	2.54	1.32–4.89	**0.005**	2.66	1.42–4.96	**0.002**	2.35	1.25–4.43	**0.008**
Grade: III vs I + II	1.89	1.04–3.46	**0.038**				1.89	1.06–3.40	**0.032**			
Stage: III + IV vs I + II	1.74	0.94–3.23	0.08				2.81	1.62–4.89	**0.001**	2.38	1.36–4.17	**0.002**
ER: (+) vs (−)	0.63	0.36–1.08	0.09				1.13	0.65–1.97	0.66			
PR: (+) vs (−)	0.59	0.34–1.03	0.06				0.79	0.45–1.38	0.40			
HER2: (+) vs (−)	0.86	0.49–1.50	0.59				0.88	0.51–1.51	0.64			
TMCC3 Tumor: high vs low	3.32	1.89–5.83	**0.001**	2.81	1.45–5.45	**0.002**	2.92	1.69–5.02	**0.001**	2.28	1.31–3.96	**0.004**

*RFS* relapse-free survival, *OS* overall survival, *HR* hazard ratio, *CI* confidence interval.

Bold values indicate statistical significance *p* < 0.05.

### Data mining confirms poor prognostic impact of high expression of *TMCC3* in breast cancer and other cancers

Using an online DNA microarray database at the ONCOMINE website, we found higher mRNA level of *TMCC3* in cancer part than normal tissue in cervical cancer, prostate cancer, pancreatic cancer, lung cancer, glioblastoma, skin cancer, hepatoma, and thyroid gland papillary carcinoma (Supplementary Fig. S6a). Using Kaplan–Meier Plotter website to evaluate the clinical significance of *TMCC3* in cancer progression, we showed that higher expression of *TMCC3* correlated with poor OS of patients with ovarian, lung, and gastric cancer (Supplementary Fig. S6b). These findings lend further support for the crucial roles of *TMCC3* in cancer progression, and for developing strategies to target TMCC3 for cancer therapy.

## Discussion

In our previous comparative phosphoproteomic analysis of BCSCs and non-BCSCs, we found a function unknown protein, TMCC3 to be expressed at higher level in BCSCs than non-BCSCs [15]. Here, we demonstrated that TMCC3 is crucial for maintaining the characteristics of BCSCs, including self-renewal, differentiation, metastasis, and tumorigenesis. In addition, we showed that TMCC3 could enhance AKT phosphorylation, which is known to promote BCSC properties.

TMCC3 was reported as an ER-anchored protein and localized at the three-way junctions of tubular ER network [20, 38]. In mouse developing embryos, *TMCC3* was expressed in the mesenchyme of developing tongue, hind brain forming tissues and lung, based on in situ-hybridization analysis [20]. However, the biological functions of TMCC3 remain enigmatic. Our study has provided the first evidence for the crucial role of TMCC3 in sustaining BCSCs properties as reflected by diminished mammosphere forming capacity, ALDH activity, in vivo metastasis and tumorigenicity upon TMCC3 silencing. In addition, we demonstrated that TMCC3 promoted AKT activation, and TMCC3 directly interacted with AKT through 1-153 a.a. fragment. This AKT-interacting domain of TMCC3 is crucial for TMCC3-induced AKT activation, mammosphere formation, and metastasis.

Dysregulation of AKT pathway is frequently observed in many type of cancers, including breast cancer, and associated with poor outcome [39, 40]. AKT activation contributes to self-renewal and differentiation of normal mammary stem cell and BCSCs [41, 42]. Mechanistically, we found TMCC3 contributes to self-renewal and metastasis by the direct binding and activation of AKT via 1-153 a.a. domain of TMCC3. In previous reports, TMCC3 was found to bind with 14-3-3 in HEK 293 cells [20]. In our mass spectrometric analysis, we did not find 14-3-3 family proteins as interacting partners in the immunoprecipitates of TMCC3 in MCF7 cells. This suggests that the interacting proteins and regulatory mechanisms of TMCC3 might vary according to the types of cells. Based on our cell-free biochemical studies, recombinant 1-158 a.a. domain of TMCC3 can directly interact with AKT without any accessory proteins. However, whether any other molecular complexes are recruited or involved in TMCC3-induced AKT activation remains to be determined.

AKT plays a center role of tumorigenesis. Upon activation, p110 PIK3CA phosphorylates phosphatidylinositol (3,4)-bisphosphate (PIP2) to form phosphatidylinositol (3,4,5)-trisphosphate (PIP3). Binding of AKT to PIP3 leads to AKT translocation from the cytoplasm to the plasma membrane in close proximity to PDK1, which phosphorylates AKT at T308 [43, 44]. The full activation of AKT requires AKT to be phosphorylated at S473 by mTORC2 [34]. Although, TMCC3 activates AKT phosphorylation at both T308 and S473, we have provided experimental evidence that PI3K, PDK1, mTORC1, or mTORC2 pathways are most likely not involved in TMCC3-induced AKT activation. Although AKT is known to be activated by PI3K/PDK1, several reports show that AKT can be activated independent of PI3K/PDK1 [45–49]. In addition, many studies have demonstrated the existence of complex crosstalks between AKT and multiple cell signaling cascades, which can further promote cancer progression and influence drug sensitivity [50]. Whether TMCC3 participates in such crosstalks awaits further investigation.

Through analysis of proteomics profiling of colorectal cancer and chronic lymphocytic leukemia, TMCC3 protein level was found to be greater in cancer parts than their normal counterparts [51, 52]. However, the clinical relevance of TMCC3 was not addressed. Our study is the first to demonstrate the prognostic significance of *TMCC3* in breast cancer. More importantly, we found that high *TMCC3* expression adversely impacted clinical outcome in early-stage luminal breast cancer. Until now, Onco*type* DX, MammaPrint, and PAM50 are the only validated biomarker assays for early-stage luminal breast cancer [53]. However, these tests are quite costly and complex. The expression level of TMCC3, if confirmed to be highly prognostic in future study, will make TMCC3 as a simple and valuable biomarker for early-stage luminal breast cancer.

In view of the adverse impact of high expression of TMCC3 on clinical outcome of breast cancer, which was corroborated by similar prognostic significance of TMCC3 in ovarian, lung and gastric cancers by data mining, and the negligible expression of *TMCC3* mRNA in human vital organs (heart, lung, liver, and kidney) based on microarray database at the ONCOMINE, TMCC3 an ideal target for the design of therapeutics to eradicate CSCs in the future.

In conclusion, we provide the first evidence supporting that TMCC3 plays important roles in maintaining BCSCs features, tumor metastasis, and tumorigenicity. TMCC3 enhances AKT activation through direct interaction with AKT. Clinically, high TMCC3 expression is an independent poor prognostic factor in breast cancer, including early-stage breast cancer. These findings support future pursuit of TMCC3 as a target for BCSCs-directed therapeutic agents.

## Materials and methods

### Cell culture

See Supplementary information for details.

### Clinical specimens

See Supplementary information for details.

### RNA extraction and qRT-PCR analysis

See Supplementary information for details.

### Mammosphere formation assay

See Supplementary information for details.

### Western blotting and immunoprecipitations

See Supplementary information for details.

### Aldefluor assay, FACS analysis, and cell sorting

See Supplementary information for details.

### Immunohistochemistry assay

See Supplementary information for details.

### Cell migration assay

See Supplementary information for details.

### Xenograft tumorigenicity

See Supplementary information for details.

### Lentiviral vector production and xCELLigence analysis

See Supplementary information for details.

### Recombinant protein production and luminex-based protein–protein interaction assay

See Supplementary information for details.

### DNA construct and DNA transfection

See Supplementary information for details.

### Statistical analysis

See Supplementary information for details.

## Supplementary information

Supplemental information

Supplementary Figure 1

Supplementary Figure 2

Supplementary Figure 3

Supplementary Figure 4

Supplementary Figure 5

Supplementary Figure 6

Supplementary Table 1

Supplementary Table 2

Supplementary Table 3
