# Supplementary material for: Transmembrane and coiled-coil domain family 3 (TMCC3) regulates breast cancer stem cell and AKT activation
Source: Oncogene. 2021 Mar 19;40(16):2858–71. doi: 10.1038/s41388-021-01729-1 (PMC8062265; doi:10.1038/s41388-021-01729-1)
Supplement: Supplementary file 1 — Supplemental information [file 41388_2021_1729_MOESM1_ESM.docx]

**Supplementary information**

**I. Supplementary figure and table legends**

**Fig. S1. Differential expression of phosphorylated TMCC3 in BCSCs vs. non-BCSCs and protein structure and sequence alignment of TMCC3.**

**a** Expression of phosphorylated TMCC3 at S216 was determined in BCSCs and non-BCSCs of BC0145 PDX tumor in two independent experiments. **b** Predicted structural domains of human TMCC3. **c** Sequence alignment of TMCC3 proteins from *Homo sapiens*, *Mus musculus*, *Rattus norvegicus*, *Gallus gallus*, and *Danio rerio*.

**Fig. S2. The knockdown efficiency of lentiviral shRNA-TMCC3 clones and overexpression of TMCC3.**

mRNA (**a**) and protein levels (**b**) of TMCC3 in AS-B145 and AS-B634 cells transduced with three clones of shRNA-TMCC3. mRNA (**c**) and protein levels (**d**) of TMCC3 in TMCC3 overexpressing MCF7.

**Fig. S3. TMCC3 contributes to AKT phosphorylation independent of PDK1 activation.**

Levels of PDK1^S241^, AKT^T308^ or ^S473^ protein in TMCC3 silenced AS-B634 and TMCC3 overexpressing AS-B634 and MCF7 were determined by western blotting.

**Fig. S4. TMCC3 knockdown suppresses cell growth**

shRNA-TMCC3 #B and shControl transduced AS-B634 cells were cultured in complete MEM cultured medium for 6 days using xCELLigence System.

**Fig. S5. The expression of truncated TMCC3 proteins in MCF7 and MB231.**

The expression of flag-tagged full-length (Flag-FL), 1-153 aa. or 154-477 aa. truncated TMCC3 proteins were examined in transfected MCF7 and MDA-MB231 (MB231) by western blot analysis.

**Fig. S6. High expression of *TMCC3* was revealed in various cancers and correlates with poor clinical outcome.**

**a** mRNA levels of *TMCC3* in normal and tumor tissues of different cancers.

**b** RFS Kaplan–Meier plots of low and high *TMCC3* expression in ovarian, lung and gastric cancers.

**Table S1. Tumor initiating frequency of ALDH^h^ or ALDH^-^ cells FACS-sorted from BC0350R1 and BC0634 PDX tumors**

**Table S2. Distribution of TNM stage and histological grade in the reported series of breast cancer patients (n = 202)**

^a^ According to the American Joint Committee on Cancer (AJCC) TNM staging system 7th edition (2010).

**Table S3. Association of *TMCC3* mRNA expression level with clinical-pathological parameters in 202 breast cancer patients.**

^a^ According to the American Joint Committee on Cancer (AJCC) TNM staging system 7th edition (2010). Statistically significant values are displayed in boldface.

**II. Experimental Procedures**

**Cell culture**

293T and MDA-MB231 cells were maintained in DMEM supplemented with 10% (v/v) fetal bovine serum. MCF7 were maintained in modified Eagle’s medium (Corning) supplemented with 10% (v/v) fetal bovine serum, 10 μg/ml human recombinant insulin (Sigma) and 1 mM sodium pyruvate (Corning). AS-B145 and AS-B634 were derived from sorted H2K^d-^CD24^-^CD44^+^ cells of BC0145 and H2K^d-^ALDH^h^ cells of BC0634 PDX tumors, respectively. They were cultured in modified Eagle’s medium containing 10% fetal bovine serum and human insulin (10 μg/ml) at 37°C with 5% CO_2_ for limited passages (≤10 passages) to facilitate *in vitro* manipulation. Tumors engrafted from AS-B145 and AS-B634 has the same performance and characteristics as their parental PDX tumors.

**Clinical specimens**

The surgically resected tumor and adjacent non-tumor tissue specimens were collected, along with relevant clinical and pathological data from 202 patients with American Joint committee on Cancer breast cancer (BC) stage I to IV were obtained from Tri-Service General Hospital (Taipei, Taiwan) and Tissue Bank of Linkou Chang Gung Memorial Hospital (Taoyuan, Taiwan). Informed consent was obtained from all subjects before their tissue were deposited. The sample were fully encoded and used under a protocol approved by the Institutional Review Board of Human Subjects Research Ethics Committee of the Tri-Service General Hospital and Chang Gung Memorial Hospital at Linkou, Taoyuan, Taiwan (Permit number: 201304321A3C502).

**RNA extraction and Quantitative RT-PCR analysis**

Total RNA was extracted using Trizol reagent and treated with an RNase-free DNase I set (Qiagen) according to the manufacturer’s protocol. First-strand cDNA was synthesized using oligo (dT)_15_ primer of reverse transcription system (Promega). Quantitative RT-PCR was using LightCycler 480 SYBR Green I Master (Roche) according to manufacturer’s instructions using LightCycler 480 II real-time PCR system (Roche). *GAPDH* was used as an internal control. For clinical sample, Glyceraldehyde-3-phosphate dehydrogenase (*GAPDH*), ubiquitin C (*UBC*) and glucuronidase beta (*GUSB*) were used as endogenous controls. The following primers were used: TMCC3, forward primer 5’-TGA GAC AGC CAA CCT GAA GCA G-3', reverse primer 5’-ATG CGA GTC TGG CAG GAT TCC A-3'; GAPDH, forward primer 5’- GTC TCC TCT GAC TTC AAC AGC G-3’, reverse primer 5’- ACC ACC CTG TTG CTG TAG CCA A-3’; UBC, forward primer 5’-ATT TGG GTC GCG GTT CTT G-3’, reverse primer 5’- TGC CTT GAC ATT CTC GAT GGT-3’; GUSB, forward primer 5’-CTC ATT TGG AAT TTT GCC GAT T-3’. Gene expression data shows normal distribution, justifying the used of Shapiro-Wilk test. No clinical qPCR result was excluded from the analysis.

**Western blotting**

Cells were lysed in RIPA buffer supplemented with phosphatase and protease inhibitors, and then boiled after mix with protein loading buffer. Prepared protein samples were loaded on SDS-PAGE, electrophoresis, and then proteins were transferred to PVDF membranes. Blots were incubated with TBS blocking buffer containing 3% BSA for 1 hour at room temperature and then with the respective primary antibodies diluted in TBST (containing 0.05% Tween20 and 3% BSA) overnight at 4 degree. Subsequently, blots were washed and incubated with appropriate secondary antibodies in TBST. Anti-TMCC3 (HPA014272) was from Sigma-ALDRICH Inc. Anti-pAKT (S473, #4058), AKT (#9272), pPDK1 (S241, #3438), PDK1 (#13037), pmTOR (S2448, #2971), mTOR (#2972), p110α (#4249), p-p85 (T458, #4228), p85 (#4257), pRictor (T1135, #3086), Rictor (#2114), pRaptor (S792, #2083), Raptor (#2280) and pSIN1 (T86, #14716) antibodies were from Cell signaling Technology. Anti-SIN1 (#05-1044) antibody was from Millipore. Anti-GAPDH (GTX100118) antibody was from GeneTex Inc.

**Mammosphere formation assay**

Single cells were plated at a density of 2,000 cells per well in ultralow attachment plates (35 mm; Corning). Cells were grown in serum-free DMEM/F12, supplemented with 0.4% BSA, B27 (1:50, Invitrogen), 20 ng/ml EGF and 20 ng/ml bFGF (BD Biosciences), and 10 ng/ml insulin (Sigma). The mammospheres were cultured for 7–10 d. Then the mammospheres with diameter >100 μm were counted.

**Aldefluor assay and flow cytometry analysis**

To measure the ALDH activity of lentiviral knocked down cells, the Aldefluor assay was performed according to manufacturer’s (Stemcell Technologies) guidelines. sh-TMCC3 and sh-Control transfected cells were suspended in Aldefluor assay buffer containing ALDH subtract, Bodipyaminoacetaldehyde (BAAA) and incubated for 30 minutes at 37 °C. To distinguish between ALDH-positive and –negative cells, a fraction of cells was incubated under identical condition in the presence of the ALDH inhibitor, diethylamino benzaldehyde (DEAB). This results in a significant decrease in the fluorescence intensity if ALDH-positive cells and was used to compensate the flow cytometer.

**DNA construct and DNA transfection**

To generate constructs of full-length or truncated TMCC3s, the following primers were used to amplify cDNA fragments: Full length: 5’-AATCTAGAATGCCGGGCAGCGACACG GCGCTCA-3’ and 5’-CCGGATCCTCATCTTGGTATTATCATCCTTTCTATGG-3’; 1-153: 5’-AATCTAGAATGCCGGGCAGCGACACGGCGCTCA-3’ and 5’-CCGGATCCTCACTTTGAGCTCCTAGAGGCTCCATTCT-3’; 1-282: 5’-AATCTAGAATGCCGGGCAGCGACACGGCGCTCA-3’ and 5’-CCGGATCCTCAGTCCAGTGTGCTGGCTCCACCAGCC-3’; 154-282: 5’-AATCTAGAGACATTTCCAAAGACCACCTGAAGG-3’ and 5’-AAGGATCCTCAGTCCAGTGTGCTGGCTCCACCA-3’;282-417: 5’-AATCTAGAAGCCAGGGCAAGCTCGCCGTGATCCT-3’ and 5’-CCGGATCCTCAGTTGATGCACCTCCCCAGGAGAA-3’; 154-477: 5’- AATCTAGA GACATTTCCAAAGACCACCTGA-3’ and 5’-CCGGATCCTCATCTTGGTATTATCATCCTTTCTATGG-3’ and cloned in the vector pFLAG-CMV-2 (#E7033, Sigma) using the restriction enzymes XbaI and BamHI to obtain the plasmids for the expression of Flag-tagged TMCC3s. All the constructs were sequenced verified. Transfections of plasmid DNA were performed with PolyJet In Vitro transfection reagent (SignaGen Laboratories) according to the manufacturer's instructions. Transfected cells were washed with PBS, and then harvested for further experiment after 48 hours post transfection.

**Lentiviral vector production and xCELLigence analysis**

TRCN0000179802 clone (targerting sequence: CCGGCGACAACATTGCTCACTTGAACTCGAGTTCAAGTGAGCAATGTTGTCGTTTTTTG), TRCN0000183750 clone (targerting sequence: CCGGCGTCATGACATGAATACCTTACTCGAGTAAGGTATTCATGTCATGACGTTTTTTG), TRCN0000180412 (targerting sequence: CCGGGATGGGAATGTTGCGGAGTATCTCGAGATACTCCGCAACATTCCCATCTTTTTTG), pLKO.1-puro-LacZ (control), pMD.G plasmid and pCMVΔR8.91 plasmid were obtained form National RNAi Core Facility at the Institute of Molecular Biology, (Academia Sinica, Taipei, Taiwan). For lentiviral transduction, cells were infected with lentiviral particles at multiplicities of infection from 1 to 10. After 24 hours infection, the culture media was replaced with MEM complete medium containing 2μg/ml puromycin for another 48 hours. The lentiviral transfected cells were collected for the further experiments and q-PCR or western blotting will be performed for checking the knockdown efficiency of shRNA-TMCC3.

shRNA transduced cells (5,000 cells/well) were plated in 96-well xCELLigence plate (RTCA E-palte, Agilent), and the cell proliferation indexs were continuously moinitored for 6 days (xCELLigence RTCA MP, Agilent).

**Immunoprecipitations**

293T cells were co-transfected with flag-tagged TMCC3 (wild type or truncated protein) and HA-tagged AKT DNA plasmids. After flag protein pull-down using anti-flag antibody, the immunoprecipitates were examined for HA-AKT proteins with anti-HA antibody by western blotting.

**Cell sorting**

To sort H2k^d-^CD24^-^CD44^+^ cells from xenograft tumor, mouse engrafted tumors were subjected to enzymatic digestion by incubation in RPMI1640 medium containing collagenase IV (1,000 U/ml), hyaluronidase (300 U/ml), and DNase I (100 μg/ml) at 37°C for 0.5 h. Primary breast tumor cells were collected and resuspended in RPMI1640 medium supplemented with 5% FBS. Cells were prepared in RPMI1640 containing 5% FBS and antibiotics (penicillin/streptomycin) and then labeled with anti-CD44-APC, anti-CD24-PE and anti-mouse H2k^d^ conjugated FITC antibody and 7AAD mixtures (BD Pharmingen). H2k^d-^CD24^-^CD44^+^ cell populations were gated and sorted out respectively by FACS Aria II cell sorter (BD Biosciences).

**Immunohistochemistry assay**

Tumor and mouse organs were collected and fixed with 4% formalin and paraffin embedded. Five μm sections were cut and mounted on microscope slides. After antigen retrieval, slides were stained with appropriate primary antibody or isotype antibody as control.

**Cell migration assay**

2x10^4^ shRNA-transduced cells were suspended in 100 μl serum-free medium, seeded in the upper insert of transwell plate (Corning Life Sciences) and then inserted into 24-well plates with 10% FBS-containing medium. Cells migrated across the membrane of the insert were stained with 0.2% crystal violet after overnight culture at 37°C. After removing the cells attached on the inner face of the insert, migrated cells were calculated and recorded by microscopy.

**Recombinant protein production and luminex-based protein–protein interaction assay**

For Luminex-based protein-protein interaction assay, 1-158 a.a. domain of *TMCC3* gene was sub-cloned into pET15b vector. *Escherichia coli* (DH5α) was transformed with 1-158 a.a. domain of TMCC3 expressing plasmid and cultured in 1.0 mM isopropyl-β-D-thiogalactoside (IPTG) at 20°C for 20 hrs. After cell lysis and sonication, recombinant 1-158 a.a. domain of TMCC3 was purified with HisTrap Excel column (GE Healthcare Biosciences) by ÄKTA protein purification systems (ÄKTA Avant 25, Cytiva). The quality of purified protein was confirmed with SDS-PAGE analysis.

Luminex immunosandwich assay was performed according to the manufacturer**’**s manual (The xMAP Cookbook, 3^rd^ Edition). Briefly, recombinant TMCC3 was coupled on beads and placed into 96-well plates. In binding assay, serial dilutions of recombinant AKT1 (#14-276, Millipore) were added to TMCC3-coupled beads, followed by detection of bound AKT with rabbit anti-AKT1 (#9272, Cell signaling) and PE-conjugated anti-rabbit IgG (#406421, Biolegend) antibodies. Recombinant Puf-A protein conjugated beads were used as control.

**Xenograft tumorigenicity and tumor metastasis**

NSG mice were purchased from the Jackson laboratory and housed at a specific-pathogen-free animal facility according to all applicable laws and regulations subsequent to approval by Chang-Gung University’s Institutional Animal Care and Use committee (Permit number: CGU106-055).

Before cell injection, mice were randomly divided into two groups. Tumor cells were mix with matrigel (356237, BD Bioscience) and then injected into the mammary fat-pads of 6-8 week old, female NSG mice. Mice were monitored weekly for the tumor growth. No blinding was done in our animal studies.

**Statistical Analysis**

The results of qRT-PCR analyses of the *TMCC3* in these samples were shown as −ΔCT, after subtracting the geometric mean of 3 reference genes, *GAPDH*, *UBC* and *GUSB*. The prognostic performance of genes was calculated with the receiver operating characteristic curve and the area under the ROC curve. The Youden index (sensitivity + specificity −1) was used to determine the optimal cut-off value for high versus low gene expression level. Survival curves were plotted with Kaplan–Meier method, by the log-rank test applied for comparison. The Cox proportional-hazards regression model was employed to evaluate the independent prognostic factors. The statistical analyses were performed with Prism 5.0 (GraphPad Software, La Jolla) and SPSS ver. 22.0 (IBM, Armonk) software.

**III. Material list**

| **Reagent** | **Source/Company** | **Catalog no.** |
| --- | --- | --- |
| **Antibodies** | | |
| anti-TMCC3 | Sigma | Cat# HPA014272 |
| anti-pAKT(S473) | Cell Signaling Technology | Cat# 4058 |
| anti-pAKT(T308) | Cell Signaling Technology | Cat# 13038 |
| anti-pPDK1(S241) | Cell Signaling Technology | Cat# 3438 |
| anti-PDK1 | Cell Signaling Technology | Cat# 3062 |
| anti-AKT | Cell Signaling Technology | Cat# 2920 |
| anti-phospho-mTOR (S2448) | Cell Signaling Technology | Cat# 2971 |
| anti-mTOR | Cell Signaling Technology | Cat# 2972 |
| anti-p110-α | Cell Signaling Technology | Cat# 4249 |
| anti-phospho-p85 (T458) | Cell Signaling Technology | Cat# 4228 |
| anti-p85 | Cell Signaling Technology | Cat# 4257 |
| anti-phospho-Rictor (T1135) | Cell Signaling Technology | Cat# 3086 |
| anti-Rictor | Cell Signaling Technology | Cat# 2114 |
| anti-phospho-Raptor (S792) | Cell Signaling Technology | Cat# 2083 |
| anti-Raptor | Cell Signaling Technology | Cat# 2280 |
| anti-phospho-SIN1 (T86) | Cell Signaling Technology | Cat# 14716 |
| anti-SIN1 | Millipore | Cat# 05-1044 |
| anti-integrin β1 | Merck | MABT409 |
| anti-GAPDH | GeneTex | Cat# GTX100118 |
| anti-Actin | Sigma-ALDRICH | Cat# A5441 |
| anti-ki67 | DAKO | Cat# M7240 |
| anti-Flag | Sigma-ALDRICH | Cat# F3165 |
| anti-Flag | Sigma-ALDRICH | Cat# F7425 |
| anti-HA | Sigma-ALDRICH | Cat# H6908 |
| anti-HA | Sigma-ALDRICH | Cat# H3663 |
| anti-CD44-APC | BD Pharmingen | Cat# 559942 |
| anti-CD24-PE | BD Pharmingen | Cat# 555428 |
| anti-H2kd-FITC | BD Pharmingen | Cat# 553565 |
| anti-rabbit-IgG-PE | Biolegend | Cat# 406421 |
| **Biological Samples** | | |
| Patient-derived xenograft tumor: BC0145 | Laboratory of Alice L. Yu | N/A |
| Patient-derived xenograft tumor: BC0350R1 | Laboratory of Alice L. Yu | N/A |
| Patient-derived xenograft tumor: BC0634 | Laboratory of Alice L. Yu | N/A |
| Human breast cancer tissue specimens (n=202) | Tri-Service General Hospital (Taipei, Taiwan) and Tissue Bank of Linkou Chang Gung Memorial Hospital (Taoyuan, Taiwan) | (Permit number: 201304321A3C502) |
| **Chemicals, Peptides, and Recombinant Proteins** | | |
| 7AAD | BD Pharmingen | Cat# 559925 |
| Matrigel | BD Pharmingen | Cat# 356237 |
| human FGF basic recombinant protein | Invitrogen | Cat# RP-8628 |
| human EGF recombinant protein | Invitrogen | Cat# RP-8661 |
| huamn insulin recombinant protein | Sigma-ALDRICH | Cat# I9278 |
| AKT1 protein | Millipore | Cat# 14-276 |
| B27 | Gibco | 17504-044 |
| DMEM | CORNING | 10-017-CV |
| MEM | CORNING | 10-010-CV |
| DMEM/F12 | Lonza | 12-719F |
| Sodium pyruvate | Gibco | 11360-070 |
| Trizol reagent | Invitrogen | 15596026 |
| RIPA Lysis and Extraction Buffer | Thermo Fisher Scientific | Cat# 89900 |
| PolyJet™ In Vitro DNA Transfection Reagent | SignaGen Laboratories | Cat# SL100688 |
| Fast SYBR Green Master Mix | Thermo Fisher Scientific | Cat# 4385612 |
| NuPAGE™ MES SDS Running Buffer (20X) | Thermo Fisher Scientific | Cat# NP0002 |
| **Critical Commercial Assays** | | |
| ALDEFLUOR™ Kit | STEMCELL | Cat# 01700 |
| Mem-PER^TM^ Plus  Membrane Protein Extraction Kit | Thermo Fisher Scientific | Cat# 89842 |
| MagPlex Microspheres | R&D | MC10022-YY |
| **Experimental Models: Cell Lines** | | |
| MDA-MB231 | BCRC | 60425 |
| MCF7 | BCRC | 60436 |
| 293T | BCRC | 60019 |
| **Experimental Models: Organisms/Strains** | | |
| NSG female mice (NOD.Cg-Prkdcscid Il2rgtm1Wjl/SzJ) | Jackson Laboratory | 005557 |
| **Oligonucleotides** | | |
| Forward primer for *TMCC3*: 5'-TGAGACAGCCAACCTGAAGCAG-3' | This paper | N/A |
| Reverse primer for *TMCC3*: 5'-ATGCGAGTCTGGCAGGATTCCA-3' | This paper | N/A |
| Forward primer for *GAPDH*: 5'-GTCTCCTCTGACTTCAACAGCG-3’ | This paper | N/A |
| Reverse primer for *GAPDH*: 5'-ACCACCCTGTTGCTGTAGCCAA-3’ | This paper | N/A |
| Forward primer for *GUSB*: 5'-CTCATTTGGAATTTTGCCGATT-3’ | This paper | N/A |
| Reverse primer for *GUSB*: 5'-CCGAGTGAAGATCCCCTTTTTA-3’ | This paper | N/A |
| Forward primer for *UBC*: 5'-ATTTGGGTCGCGGTTCTTG-3’ | This paper | N/A |
| Reverse primer for *UBC*: 5'-TGCCTTGACATTCTCGATGGT-3’ | This paper | N/A |
| **Recombinant DNA** | | |
| TRCN0000179802 | National RNAi Core Facility at the Institute of Molecular Biology, (Academia Sinica, Taipei, Taiwan) | http://rnai.genmed.sinica.edu.tw/index |
| TRCN0000183750 | National RNAi Core Facility at the Institute of Molecular Biology, (Academia Sinica, Taipei, Taiwan) | http://rnai.genmed.sinica.edu.tw/index |
| TRCN0000180412 | National RNAi Core Facility at the Institute of Molecular Biology, (Academia Sinica, Taipei, Taiwan) | http://rnai.genmed.sinica.edu.tw/index |
| pLKO.1-puro-LacZ | National RNAi Core Facility at the Institute of Molecular Biology, (Academia Sinica, Taipei, Taiwan) | http://rnai.genmed.sinica.edu.tw/index |
| pMD.G | National RNAi Core Facility at the Institute of Molecular Biology, (Academia Sinica, Taipei, Taiwan) | http://rnai.genmed.sinica.edu.tw/index |
| pCMVΔR8.91 | National RNAi Core Facility at the Institute of Molecular Biology, (Academia Sinica, Taipei, Taiwan) | http://rnai.genmed.sinica.edu.tw/index |
| pFLAG-CMV-2-TMCC3-FL | This paper | N/A |
| pFLAG-CMV-2-TMCC3-1-153aa. | This paper | N/A |
| pFLAG-CMV-2-TMCC3-154-477aa. | This paper | N/A |
| pFLAG-CMV-2-TMCC3-1-282aa. | This paper | N/A |
| pFLAG-CMV-2-TMCC3-154-282aa. | This paper | N/A |
| pFLAG-CMV-2-TMCC3-283-416aa. | This paper | N/A |
| pcDNA3.1-HA-AKT | This paper | N/A |
| pET15b vector | Sigma-ALDRICH | Cat# 69661 |
| **Software and Algorithms** | | |
| GraphPad Prism 5.0 | GraphPad Software | N/A |
| ImageQuant 5.2 | GE Healthcare | N/A |
| Tissue Studio | Definiens | N/A |
| SPSS ver. 22.0 | IBM | N/A |
| FLOWJO | BD | N/A |
