# Supplementary figures and images for: Transmembrane and coiled-coil domain family 3 (TMCC3) regulates breast cancer stem cell and AKT activation
Source: Oncogene. 2021 Mar 19;40(16):2858–71. doi: 10.1038/s41388-021-01729-1 (PMC8062265; doi:10.1038/s41388-021-01729-1)

# Supplementary Figure 1

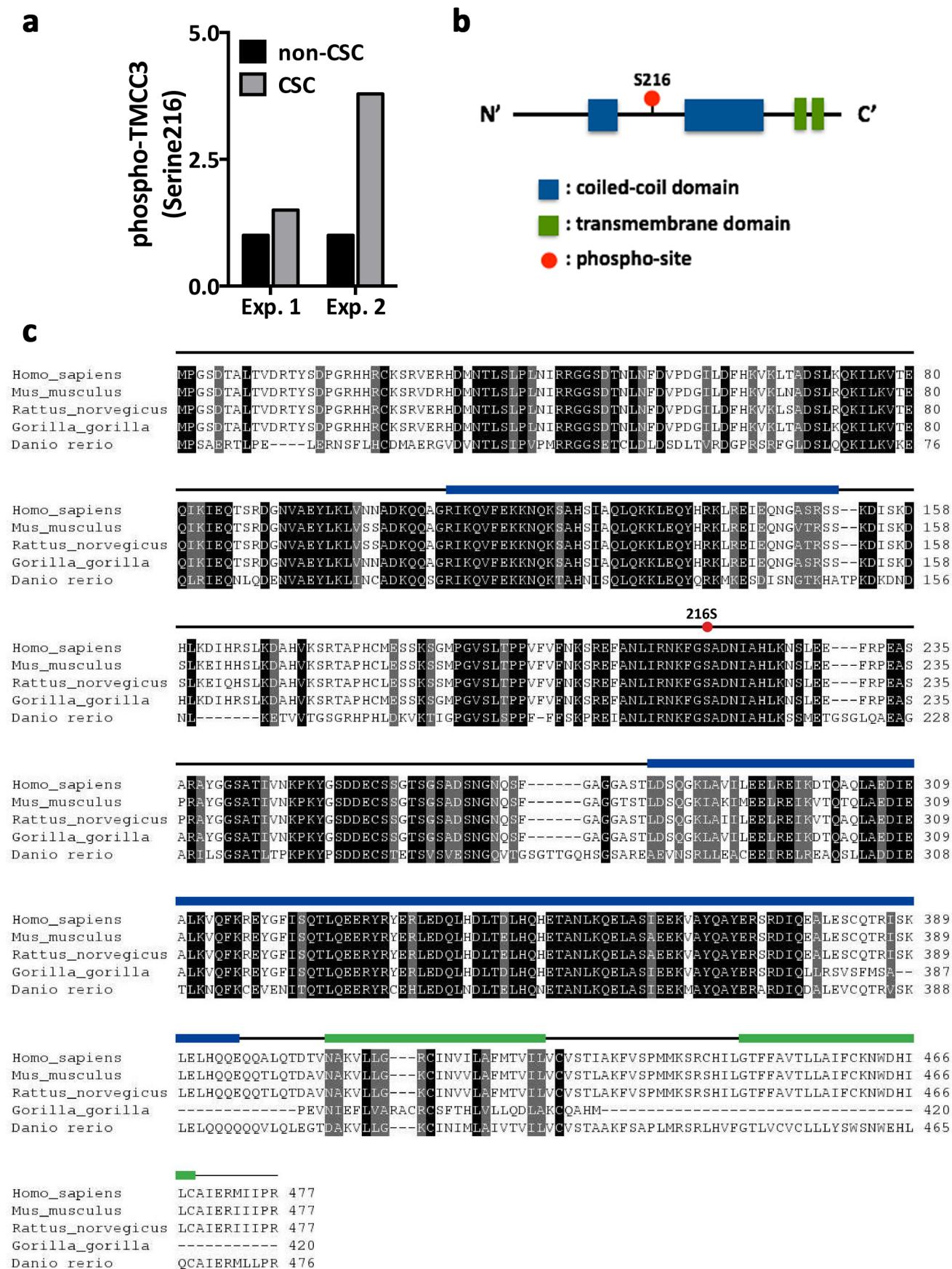

Supplement: Supplementary file 2 — Supplementary Figure 1 [file 41388_2021_1729_MOESM2_ESM.pdf]

## Supplementary Figure 2

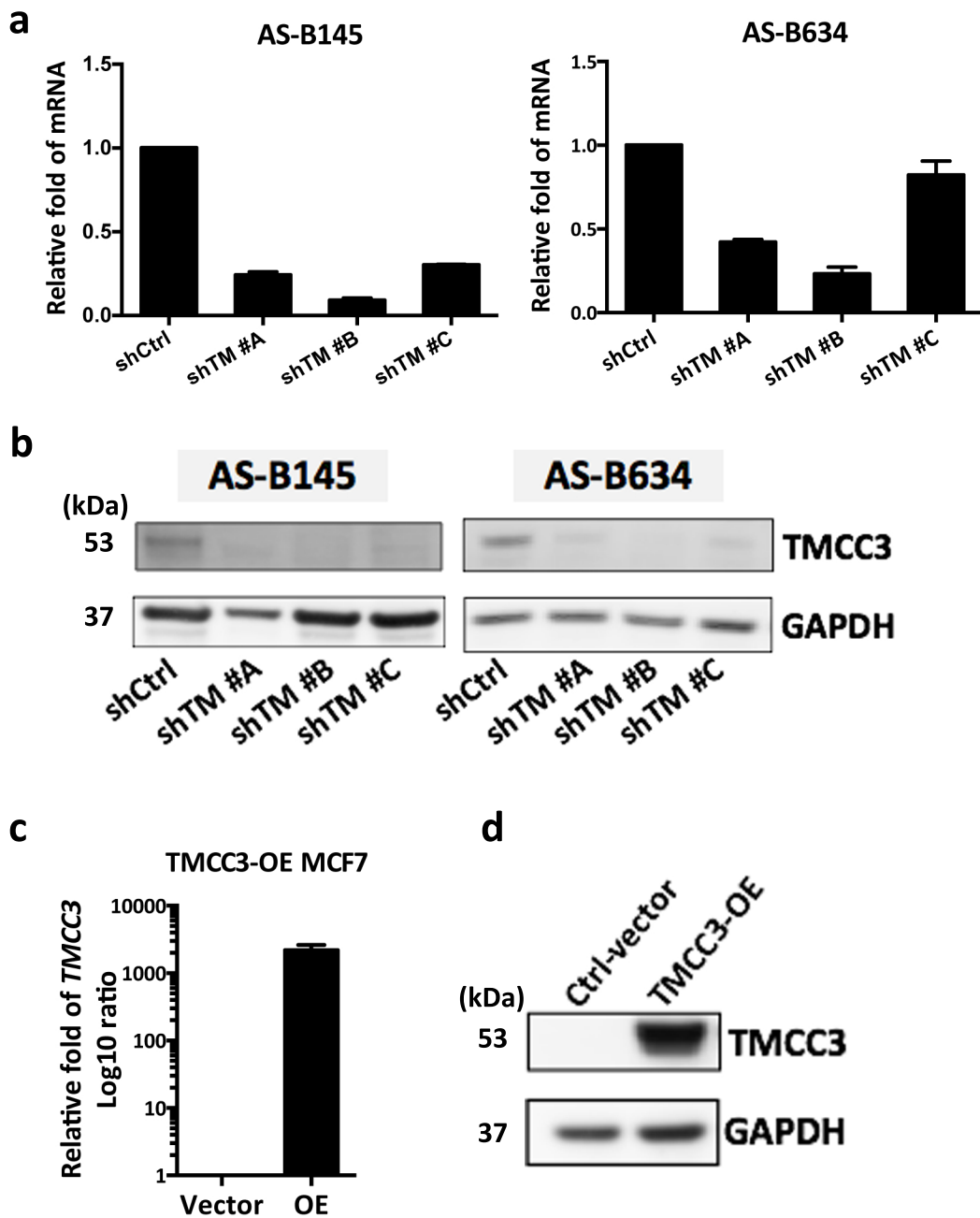

Supplement: Supplementary file 3 — Supplementary Figure 2 [file 41388_2021_1729_MOESM3_ESM.pdf]

# Supplementary Figure 3

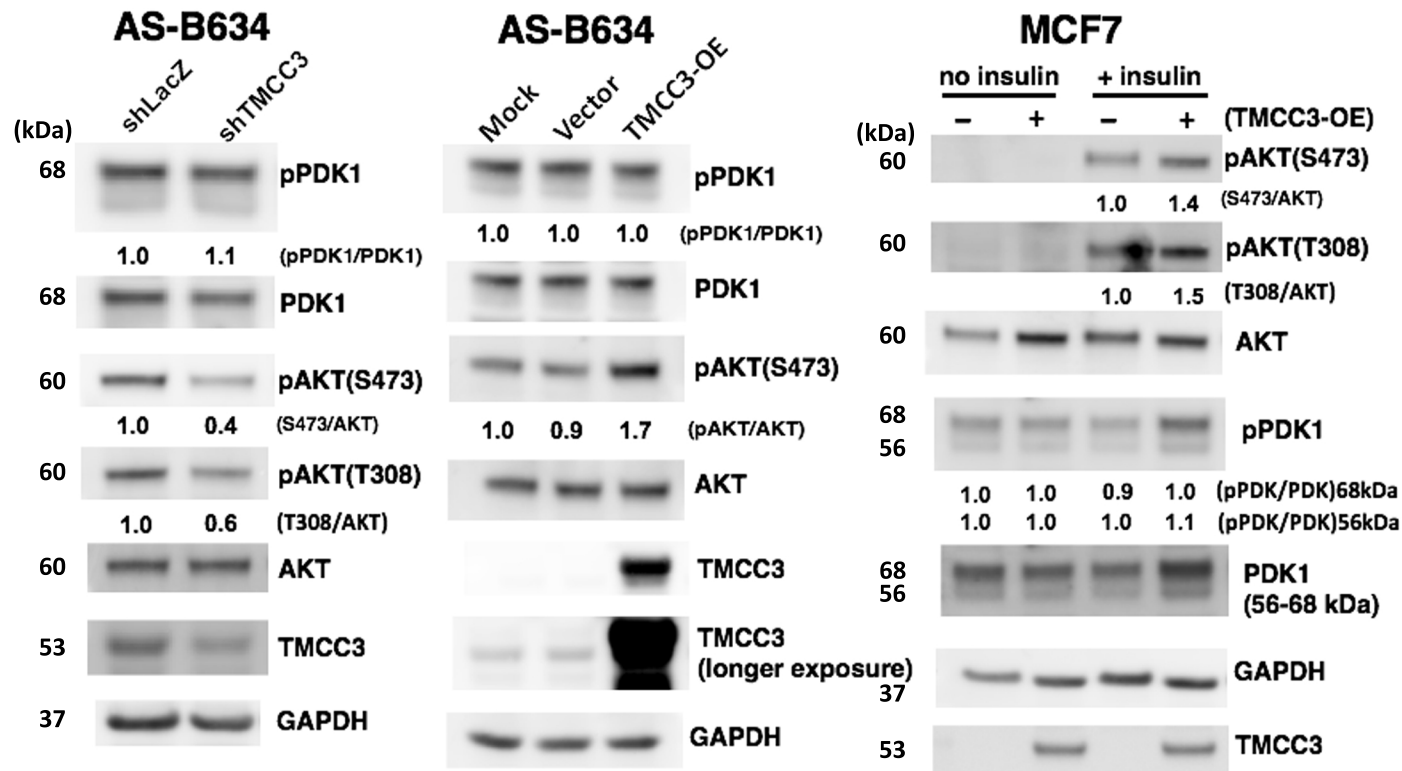

Supplement: Supplementary file 4 — Supplementary Figure 3 [file 41388_2021_1729_MOESM4_ESM.pdf]

Supplementary Figure 4

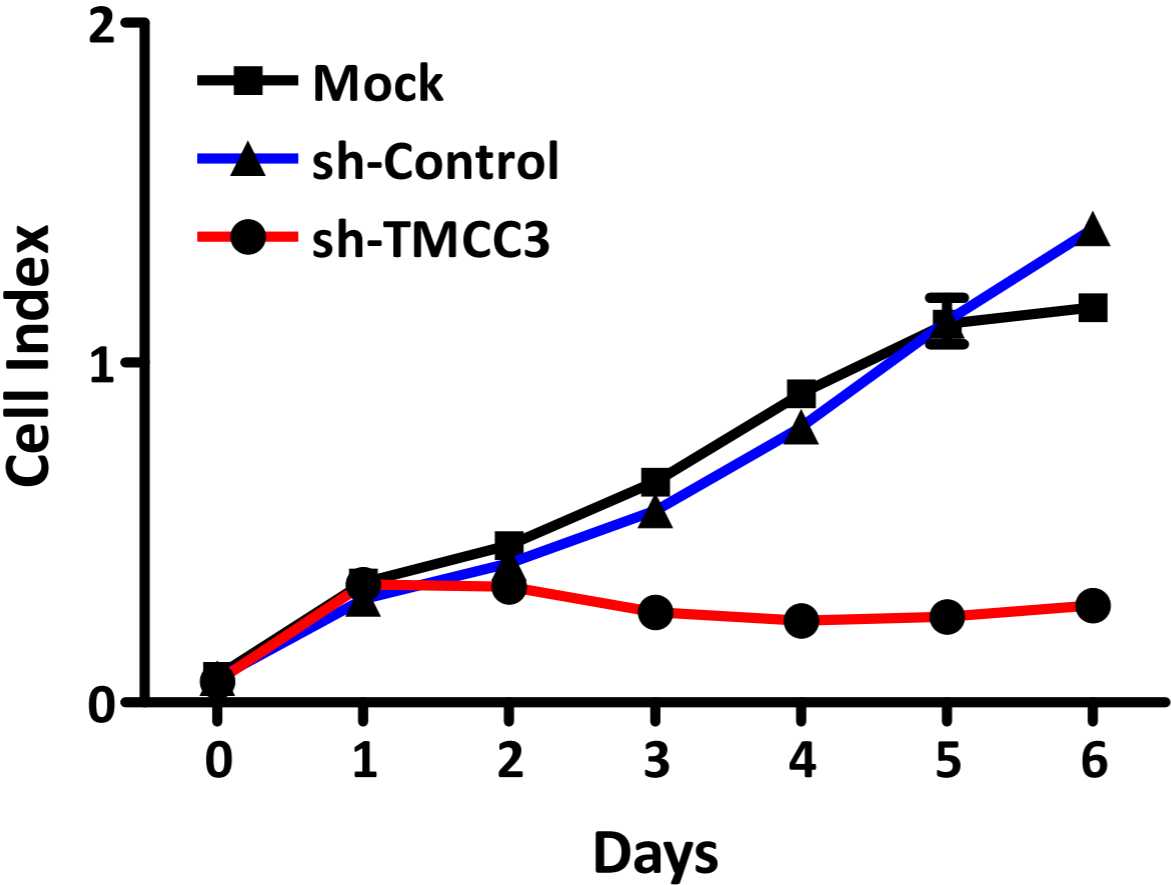

Supplement: Supplementary file 5 — Supplementary Figure 4 [file 41388_2021_1729_MOESM5_ESM.pdf]

# Supplementary Figure 5

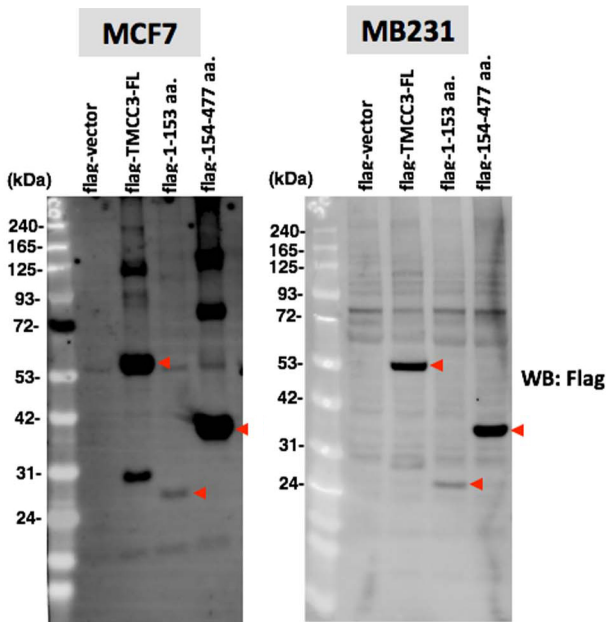

Supplement: Supplementary file 6 — Supplementary Figure 5 [file 41388_2021_1729_MOESM6_ESM.pdf]

# Supplementary Figure 6

a

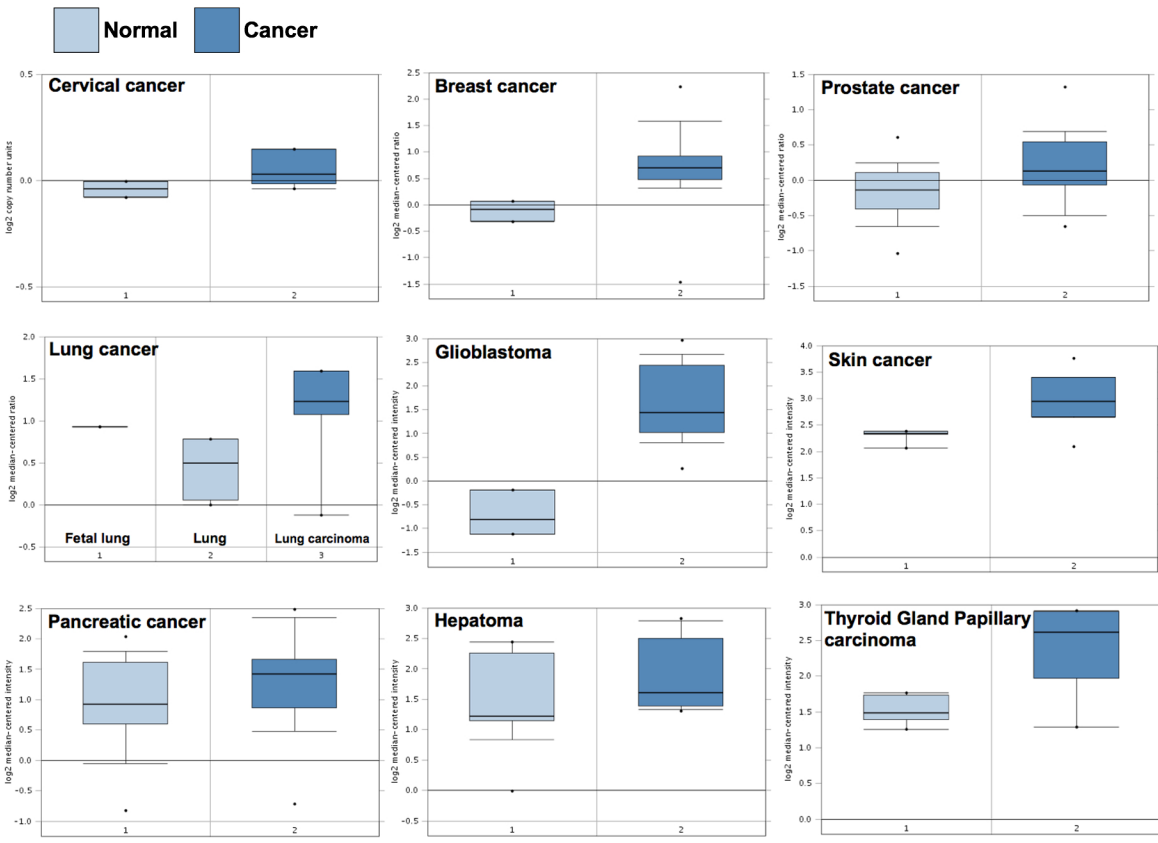

b

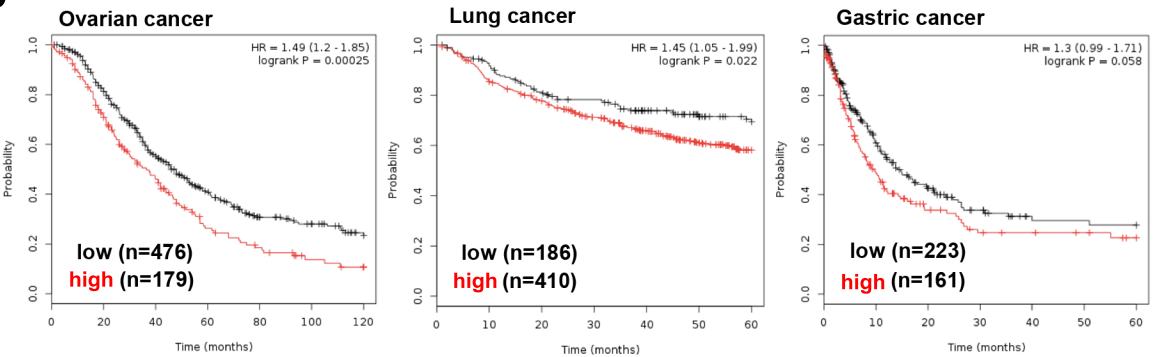

Supplement: Supplementary file 7 — Supplementary Figure 6 [file 41388_2021_1729_MOESM7_ESM.pdf]
