# Supplementary material for: Transmembrane and coiled-coil domain family 3 (TMCC3) regulates breast cancer stem cell and AKT activation
Source: Oncogene. 2021 Mar 19;40(16):2858–71. doi: 10.1038/s41388-021-01729-1 (PMC8062265; doi:10.1038/s41388-021-01729-1)
Supplement: Supplementary file 8 — Supplementary Table 1 [file 41388_2021_1729_MOESM8_ESM.docx]

Supplementary Table 1

|  |  |  | **(Injection cell no.)** | | | | | |  |  |
| --- | --- | --- | --- | --- | --- | --- | --- | --- | --- | --- |
| **PDX code** | **Diagnosis (Grade)** | **CSC marker** | **2x10^5^** | **1x10^5^** | **2x10^4^** | **1x10^4^** | **2x10^3^** | **1x10^3^** | **CSC frequency** | **P value** |
| **BC0350R1** | **Recurrent IDC (III)** |  |  |  |  |  |  |  |  |  |
|  |  | **ALDH^h^** |  | **3/3** |  | **2/3** |  | **2/3** | **1:4,659** | **0.0004** |
|  |  | **ALDH^-^** |  | **1/3** |  | **2/3** |  | **0/3** | **1:87,841** |  |
| **BC0634** | **IDC (III)** |  |  |  |  |  |  |  |  |  |
|  |  | **ALDH^h^** | **3/3** | **2/2** | **2/3** | **2/3** | **2/3** | **0/3** | **1:9,816** | **0.0006** |
|  |  | **ALDH^-^** | **3/3** | **0/2** | **1/3** | **1/3** | **0/3** | **0/3** | **1:97,512** |  |
