# Supplementary material for: Transmembrane and coiled-coil domain family 3 (TMCC3) regulates breast cancer stem cell and AKT activation
Source: Oncogene. 2021 Mar 19;40(16):2858–71. doi: 10.1038/s41388-021-01729-1 (PMC8062265; doi:10.1038/s41388-021-01729-1)
Supplement: Supplementary file 9 — Supplementary Table 2 [file 41388_2021_1729_MOESM9_ESM.docx]

Supplementary Table 2

| **Characteristics** | **N (%)** |
| --- | --- |
| Age (Mean ± SD and range) (years) | 54.4 ± 12.2 (30-89) |
| Grade |  |
| 1 | 10 (5%) |
| 2 | 73 (36.1%) |
| 3 | 119 (58.9%) |
| Stage^a^ |  |
| I | 67 (33.2%) |
| II | 94 (46.5%) |
| III | 39 (19.3%) |
| IV | 2 (1%) |
| Tumor stage |  |
| pT1 | 93 (46%) |
| pT2 | 93 (46%) |
| pT3 | 13 (6.5%) |
| pT4 | 3 (1.5%) |
| Lymph nodes |  |
| pN0 | 116 (57.4%) |
| pN1 | 52 (25.7%) |
| pN2 | 16 (8.0%) |
| pN3 | 18 (8.9%) |
| Relapse (Mean ± SD and range) (years) | 2.14  ± 1.71 (0.30-8.84) |
| No | 151 (74.3%) |
| Yes | 51 (25.7%) |
| Death (Mean ± SD and range) (years) | 5.00 ± 3.10 (0.44-11.86) |
| No | 148 (73.3%) |
| Yes | 54 (26.7%) |
