# Supplementary material for: Transmembrane and coiled-coil domain family 3 (TMCC3) regulates breast cancer stem cell and AKT activation
Source: Oncogene. 2021 Mar 19;40(16):2858–71. doi: 10.1038/s41388-021-01729-1 (PMC8062265; doi:10.1038/s41388-021-01729-1)
Supplement: Supplementary file 10 — Supplementary Table 3 [file 41388_2021_1729_MOESM10_ESM.docx]

Supplementary Table 3

|  | Variable | TMCC3 tumor　(n=202) | | | | |  |
| --- | --- | --- | --- | --- | --- | --- | --- |
|  |  | N | Low | High | P value | OR (95% CI) |  |
|  | Age | | | | | |  |
|  | <50 | 86 | 61 | 25 | 0.18 | 1.55 (0.85-2.81) |  |
|  | ≥50 | 116 | 71 | 45 |  |  |  |
|  | Grade |  |  |  |  |  |  |
|  | G1+G2 | 83 | 59 | 24 | 0.18 | 1.55 (0.85-2.83) |  |
|  | G3 | 119 | 73 | 46 |  |  |  |
|  | Stage^a^ | | | | | |  |
|  | I + II | 161 | 109 | 52 | 0.20 | 1.64 (0.82-3.30) |  |
|  | III + IV | 41 | 23 | 18 |  |  |  |
|  | Tumor stage | | | | | |  |
|  | pT1+2 | 186 | 121 | 65 | 1.00 | 0.85 (0.28-2.54) |  |
|  | pT3+4 | 16 | 11 | 5 |  |  |  |
|  | Lymph nodes | | | | | |  |
|  | Negative | 116 | 83 | 33 | **0.04** | 1.89 (1.06-3.42) |  |
|  | Positive | 86 | 49 | 37 |  |  |  |
|  | ER | | | | | |  |
|  | Negative | 83 | 50 | 33 | 0.23 | 0.48 (0.23-1.01) |  |
|  | Positive | 119 | 82 | 37 |  |  |  |
|  | PR | | | | | |  |
|  | Negative | 68 | 49 | 29 | 0.12 | 0.59 (0.32-1.09) |  |
|  | Positive | 134 | 93 | 41 |  |  |  |
|  | HER2 | | | | | |  |
|  | Negative | 113 | 70 | 43 | 0.29 | 0.71 (0.39-1.28) |  |
|  | Positive | 89 | 62 | 27 |  |  |  |
|  | Relapse |  |  |  |  |  |  |
|  | No | 151 | 112 | 39 | **<0.001** | 4.45 (2.27-8.69) |  |
|  | Yes | 51 | 20 | 31 |  |  |  |
|  | Death |  |  |  |  |  |  |
|  | No | 148 | 109 | 39 | **<0.001** | 3.77 (1.96-7.23) |  |
|  | Yes | 54 | 23 | 31 |  |  |  |
